# Architecture and regulatory functions of c-di-GMP signaling in classical *Bordetella* species

**DOI:** 10.1093/femsre/fuaf065

**Published:** 2025-12-26

**Authors:** Denisa Vondrova, Sabrina Laura Mugni, Jan Blumenstein, Clara Kasiztky, Federico Sisti, Julieta Fernández, Jana Kamanova

**Affiliations:** Laboratory of Infection Biology, Institute of Microbiology of the Czech Academy of Sciences, Prague 142 00, Czech Republic; Instituto de Biotecnología y Biología Molecular (IBBM)-CCT La Plata, CONICET. Departamento de Ciencias Biológicas, Facultad de Ciencias Exactas, Universidad Nacional de La Plata, La Plata 1900, Argentina; Laboratory of Infection Biology, Institute of Microbiology of the Czech Academy of Sciences, Prague 142 00, Czech Republic; Instituto de Biotecnología y Biología Molecular (IBBM)-CCT La Plata, CONICET. Departamento de Ciencias Biológicas, Facultad de Ciencias Exactas, Universidad Nacional de La Plata, La Plata 1900, Argentina; Instituto de Biotecnología y Biología Molecular (IBBM)-CCT La Plata, CONICET. Departamento de Ciencias Biológicas, Facultad de Ciencias Exactas, Universidad Nacional de La Plata, La Plata 1900, Argentina; Instituto de Biotecnología y Biología Molecular (IBBM)-CCT La Plata, CONICET. Departamento de Ciencias Biológicas, Facultad de Ciencias Exactas, Universidad Nacional de La Plata, La Plata 1900, Argentina; Laboratory of Infection Biology, Institute of Microbiology of the Czech Academy of Sciences, Prague 142 00, Czech Republic

**Keywords:** c-di-GMP, *Bordetella*, *B. pertussis*, biofilm, motility, type III secretion system

## Abstract

Cyclic di-GMP (c-di-GMP) is a highly conserved bacterial second messenger that regulates important processes such as motility, biofilm formation and virulence. In this review, we investigate the architecture and regulatory functions of c-di-GMP signaling in classical *Bordetella* species, including *B. bronchiseptica, B. parapertussis* and *B. pertussis*. We examine how the c-di-GMP signaling pathway interacts with the BvgAS two-component system and other signaling pathways to coordinate virulence gene expression and surface-associated behaviors in these respiratory pathogens. In particular, we highlight the functions of characterized diguanylate cyclases (DGCs), phosphodiesterases (PDEs) and dual-domain proteins, focusing on regulatory modules such as the BdcA-DdpA scaffold complex, the oxygen-sensing DGC *Bpe*GReg and the LapD-LapG proteolytic switch that controls BrtA adhesin. We also propose a model for the function of BvgR, a PDE-like protein lacking catalytic residues, and discuss how c-di-GMP suppresses the type III secretion system. Importantly, we highlight the diversity of the c-di-GMP network in classical *Bordetella* species, likely reflecting their evolutionary specialization. To conclude, we outline important open questions and suggest future research directions, including the identification of sensory ligands and c-di-GMP effectors. Overall, our review illustrates the importance of c-di-GMP as a critical, but still incompletely understood, regulatory hub in *Bordetella* pathogenesis.

## Teaser

This review summarizes current literature on c-di-GMP signaling in classical *Bordetella* species and examines the molecular architecture of the network and its regulatory roles in *Bordetella* physiology and pathogenesis.

## Introduction

Bacterial pathogens have evolved finely tuned signal transduction systems that enable them to recognize environmental cues and adapt to changing conditions during host infection (Miller et al. 1989). Cellular processes such as virulence gene expression, motility and metabolism are tightly regulated and respond to signals including the availability of amino acids and sugars, the iron deficiency, and the presence of host hormones and other molecules. These diverse stimuli are integrated by networks of signaling cascades involving membrane-bound sensor proteins, two-component regulatory systems (TCSs), second messengers and downstream transcription factors that coordinate gene expression (Randall et al. 2022, Chen et al. 2023, Matilla et al. 2025).

The same principles apply to the so-called classical *Bordetella* species, which include *B. pertussis, B. parapertussis*, and *B. bronchiseptica*. These closely related Gram-negative pathogens cause respiratory infections in humans and animals, although they differ in their host range and disease manifestations. *B. pertussis* is a strictly human-adapted pathogen that lacks an animal reservoir and cannot survive outside the host. It is the causative agent of whooping cough, also known as pertussis, a severe and sometimes fatal respiratory disease, especially in unvaccinated infants (Mattoo and Cherry 2005). Despite the availability of vaccines, pertussis remains a major public health concern worldwide. Historically, the introduction of whole-cell pertussis vaccines in the mid-20th century led to a dramatic decline in the number of pertussis cases. However, by the end of the century, a resurgence was observed in several countries. Multiple non-mutually exclusive factors have been proposed to explain this increase, including the switch from whole-cell to acellular pertussis vaccines in some countries, improved molecular detection methods and the emergence of variant circulating strains (Domenech de Celles and Rohani 2024, Wang et al. 2025). During the COVID-19 pandemic, social distancing and reduced human contact led to a marked decrease in pertussis incidence, highlighting the importance of person-to-person transmission in the pathogenesis of the disease. Nevertheless, in the years following the pandemic, several countries with acellular vaccine-based immunization programs have reported an alarming rise in pertussis cases (Khalil et al. 2024, Wang et al. 2025).

In contrast to *B. pertussis, B. bronchiseptica* primarily infects animals, such as pigs, dogs and cats, and can persist in environmental reservoirs (Goodnow 1980, Porter and Wardlaw 1993, Hamidou Soumana et al. 2017). It causes infections ranging from fatal pneumonia to asymptomatic carriage. Human infections with *B. bronchiseptica* are rare and typically limited to immunocompromised individuals (Gueirard et al. 1995, Redelman-Sidi et al. 2011, El Khatib et al. 2015). This species is considered the ancestral form from which the other two species, *B. pertussis* and *B. parapertussis*, have independently evolved through successive gene loss events (Parkhill et al. 2003, Diavatopoulos et al. 2005). This reductive evolution is associated with their host adaptation and a more specialized pathogenic lifestyle in *B. pertussis*. Interestingly, *B. parapertussis* consists of two distinct lineages, one infecting humans, *B. parapertussis*_HU_, and the other one infecting sheep, *B. parapertussis*_ov_ (Porter et al. 1994, van der Zee et al. 1997, Hester et al. 2015).

**Figure 1.**
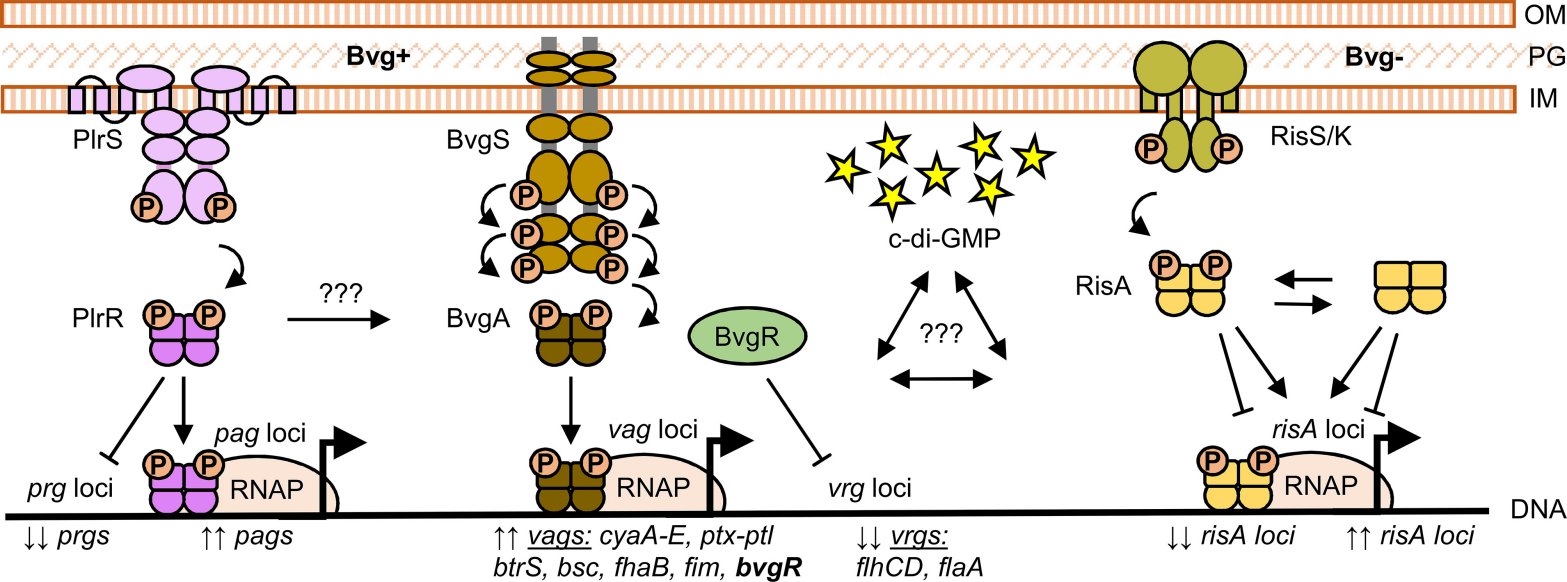
Regulation of virulence genes in classical *Bordetella* species by two-component regulatory systems BvgAS, PlrSR and RisAS/K. The master regulator of virulence gene expression in classical *Bordetella* species is the BvgAS two-component system, composed of the sensor kinase BvgS and the response regulator BvgA. In the virulent Bvg^+^ phase, BvgS is autophosphorylated and transfers the phosphate group to BvgA, which then activates the transcription of *vags* (*vir*-activated genes), including those encoding toxins and adhesins. In contrast, *vrgs* (*vir*-repressed genes) are expressed only at very low levels in the Bvg^+^ phase. Their repression is mediated by BvgR, a transcriptional regulator whose own expression is BvgA-dependent. The PlrSR system supports BvgS activity, likely by regulating the expression of one or more BvgAS-independent genes, termed *pags* (PlrSR-activated genes) and *prgs* (PlrSR-repressed genes). However, the PlrSR regulon remains to be fully defined. A third system called RisAS/K, consisting of the response regulator RisA and either RisS in *B. bronchiseptica* or RisK in *B. pertussis*, is essential for *vrg* expression under Bvg^-^ phase conditions. RisA functions mainly in its phosphorylated form, but also regulates a subset of genes independently of phosphorylation. RisA binds to the promoter regions of most *vrgs* and surprisingly also to some *vags*, suggesting regulatory crosstalk with BvgAS. Expression of virulence genes is further modulated by the secondary messenger c-di-GMP, which adds another layer of complexity to the regulatory network. OM, outer membrane; PG, peptidoglycan; IM, inner membrane.

Although the pathogenic potential and host range of the classical *Bordetella* species differ, they share an almost identical set of virulence factors, with the exception of pertussis toxin, which is uniquely produced by *B. pertussis* (Parkhill et al. 2003). The expression of these factors is primarily regulated by TCSs, as shown in Fig. 1. Among them, the BvgAS system is recognized as the master regulator of virulence in classical *Bordetella* species. However, over the past 15 years, accumulating evidence has demonstrated that *Bordetella* virulence regulation is more intricate than previously thought. In addition to BvgAS, other TCSs, such as PlrSR and RisAS/K, and signaling pathways mediated by the second messenger cyclic di-GMP (c-di-GMP) have emerged as key regulatory systems.

In this review, we dissect the architecture and regulatory functions of c-di-GMP signaling in classical *Bordetella* species. We begin with a brief overview of *Bordetella* TCSs, followed by a comprehensive analysis of the molecular components that constitute the c-di-GMP network. We then examine how these components integrate into the broader regulatory landscape, and contribute to the regulation of *Bordetella* virulence and surface-associated behaviors, such as biofilm formation and motility. Finally, we highlight key unanswered questions and current gaps in our understanding of how c-di-GMP signaling contributes to *Bordetella* pathogenesis.

## Two-component regulatory systems of classical *Bordetella* species

The best-characterized regulatory system in classical *Bordetella* species is the BvgAS two-component system, composed of the sensor kinase BvgS and the response regulator BvgA. BvgAS functions as the master regulator of virulence (Weiss et al. 1983, Knapp and Mekalanos 1988, Arico et al. 1989, Uhl and Miller 1994). Unlike most sensor kinases that require activation by an external signal, BvgS is constitutively active under standard laboratory conditions at 37°C in the absence of negative modulators (Lacey 1960, Herrou et al. 2010, Dupre et al. 2015). This phase, referred to as the virulent Bvg^+^ phase, is necessary for full virulence in mammalian hosts (Cotter and Miller 1994, 1997, Martinez de Tejada et al. 1998, Nicholson et al. 2012).

In the Bvg^+^ phase, as schematically illustrated in Fig. 1, phosphorylated BvgA (BvgA∼P) activates the transcription of *vir*-activated genes (*vags*, BvgA was originally called *vir*) by binding to their promoter regions. The *vags* include adhesins such as *fhaB* (filamentous hemagglutinin) and *fim* (fimbriae) as well as toxins such as *cyaA* (adenylate cyclase toxin) and *ptx* (pertussis toxin) (Hot et al. 2003, Cummings et al. 2006, Melvin et al. 2014). Additionally, BvgA∼P activates the expression of the extracytoplasmic function (ECF) sigma factor *btrS* (also referred to as *brpL*), which in turn induces the expression of type III secretion system genes located within the *bsc* locus (Moon et al. 2017). In contrast, exposure to millimolar concentrations of nicotinic acid or MgSO_4_ shifts BvgS into the kinase-off state, initiating the Bvg^-^ phase. During this phase, expression of *vags* is repressed, while *vir*-repressed genes (*vrgs*), which include genes involved in biosynthesis, metabolism, capsule formation, and motility (e.g. flagella-related genes such as *flaA*), are upregulated (Akerley and Miller 1993, Hot et al. 2003, Cummings et al. 2006, Melvin et al. 2014). In *B. bronchiseptica*, the Bvg⁻ phase facilitates environmental survival, particularly under nutrient-limiting conditions and promotes interaction with protozoa (Cotter and Miller 1994, Taylor-Mulneix et al. 2017, Nugraha et al. 2023). In contrast, in *B. pertussis*, this phase may be partially adopted in response to the harsh intracellular environment encountered within macrophages shortly after internalization (Farman et al. 2023). Importantly, the repression of certain *vrgs*, including those involved in motility, is mediated by BvgR, a regulatory protein whose expression is activated by BvgA∼P (Merkel and Stibitz 1995, Merkel et al. 2003, Fernandez et al. 2005, Merkel et al. 1998a), as shown in Fig. 1.

In addition to the Bvg^+^ and Bvg^-^ phases, *Bordetella* can adopt an intermediate regulatory state, referred to as Bvg^i^ phase. This phase is induced by lower concentrations of chemical modulators and is characterized by a distinct transcriptional profile that differs from both the fully active and fully repressed states (Cotter and Miller 1997, Deora et al. 2001). These observations led to the formulation of the so-called “rheostat model” of regulation, in which BvgS functions as a molecular rheostat that adjusts intracellular levels of BvgA∼P in response to the intensity of environmental signal. This, in turn, enables differential gene expression based on the affinity of target promoters for BvgA∼P (Mattoo et al. 2001, Cotter and Jones 2003, Chen and Stibitz 2019). Nevertheless, the identity of the *in vivo* ligands and/or regulatory proteins that are capable of attenuating BvgS activity remains elusive.

Recent evidence further indicates that BvgAS operates within a broader regulatory network that includes additional TCSs (see Fig. 1). One such system is PlrSR (Persistence in the Lower Respiratory tract, Sensor kinase and Response regulator), which belongs to the NtrYX family, and plays a critical role in supporting bacterial persistence and maintaining BvgAS activity in the lower respiratory tract (Kaut et al. 2011, Bone et al. 2017). PlrS has been hypothesized to function as an CO_2_ or oxygen sensor that undergoes autophosphorylation, and subsequently transfers the phosphate group to its response regulator PlrR. Phosphorylated PlrR then in turn regulates BvgAS-independent genes necessary for sustaining BvgS activity during colonization of the lower respiratory tract (Bone et al. 2017, Sobran and Cotter 2019, Barr et al. 2023).

The BvgAS and PlrSR systems are complemented by a third TCS known as RisAS/K, as illustrated in Fig. 1. This system consists of the response regulator RisA and a sensor kinase RisS in *B. bronchiseptica* and RisK in *B. pertussis*. In *B. pertussis*, the *risS* gene, located adjacent to *risA*, was inactivated by a frameshift mutation. As a result, RisK, a functionally redundant sensor kinase, functions as its replacement (Coutte et al. 2016, Chen et al. 2017). RisAS/K was first characterized for its role in oxidative stress resistance and intracellular survival in *B. bronchiseptica* (Jungnitz et al. 1998, Zimna et al. 2001). Later studies demonstrated that expression and phosphorylation of RisA occur independently of BvgAS. Importantly, although phosphorylated RisA is required for the expression of *vrgs*, it is not sufficient on its own, indicating that additional signals are necessary (Croinin et al. 2005, Stenson et al. 2005, Chen et al. 2017). Recent transcriptomic and ChIP-seq analyzes further revealed that RisA regulates not only the majority of *vrgs*, but also a subset of *vags*, genes outside the BvgAS regulon and several small non-coding RNAs. Moreover, some regulatory functions of RisA appear to be independent of its phosphorylation status, suggesting a more complex role within the regulatory network (Coutte et al. 2016, Keidel et al. 2018, Coutte et al. 2024, Nicholson et al. 2024).

Together, these three systems, BvgAS, PlrSR, and RisAS/K, form an interconnected network that enables classical *Bordetella* species to adapt to a wide range of host- and environment-derived signals.

## Principles of c-di-GMP signaling in bacteria

**Figure 2.**
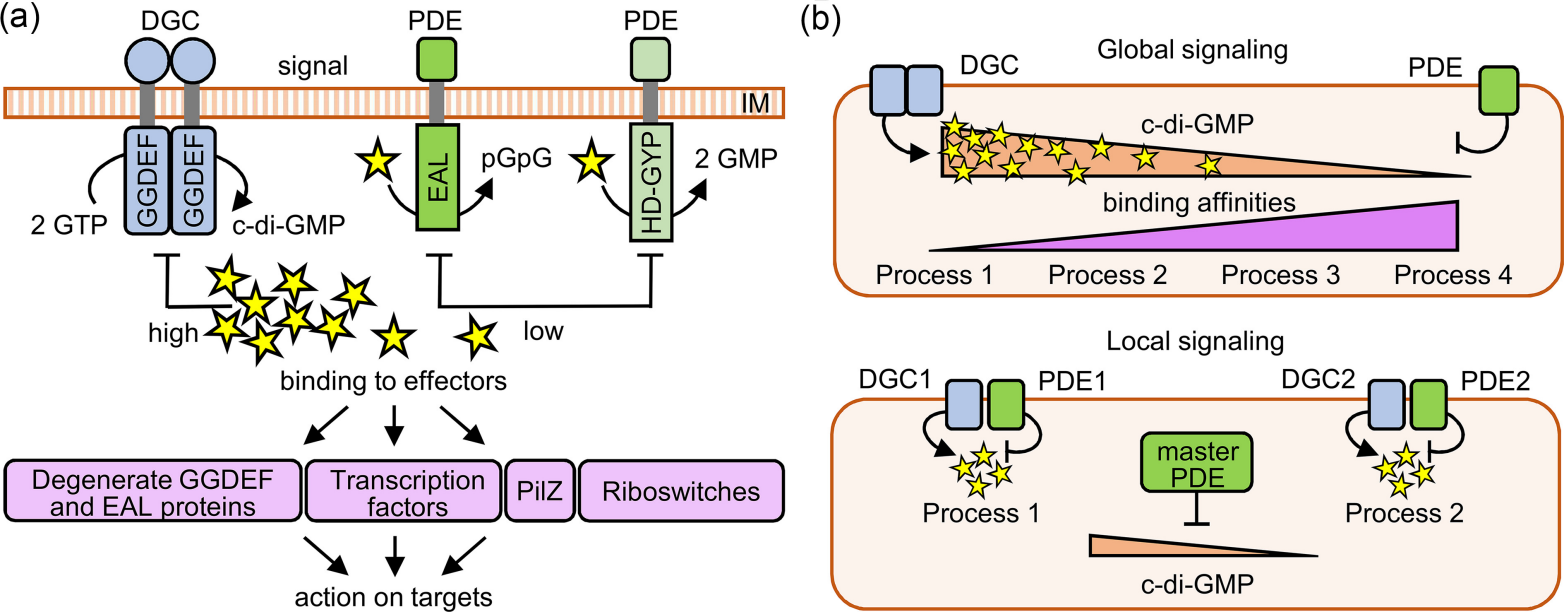
General principles of c-di-GMP signaling. **(A)** The bacterial second messenger c-di-GMP is synthesized from GTP by diguanylate cyclases (DGCs) containing GGDEF domains and degraded by phosphodiesterases (PDEs) with EAL or HD-GYP domains. Intracellular c-di-GMP levels are sensed by variety of effectors, including degenerate GGDEF/EAL domain proteins, transcription factors, PilZ domain proteins and riboswitches. These effectors act on downstream targets to modulate motility, adhesion, biofilm formation and virulence gene expression. IM, inner membrane. **(B)** Specificity of c-di-GMP signaling. In global signaling, changes in the overall cytoplasmic c-di-GMP concentration affect target systems in a hierarchical manner according to their binding affinities. In contrast, local signaling is mediated through specific protein–protein interactions between defined DGCs/PDEs and their cognate effectors, enabling spatially restricted signaling modules with distinct outputs.

C-di-GMP (cyclic-di-GMP or bis-(3’-5’)-cyclic-dimeric guanosine monophosphate) is a ubiquitous second messenger, first identified in 1987, that regulates a broad spectrum of bacterial processes, including motility, biofilm formation and virulence (Ross et al. 1987). The intracellular concentration of c-di-GMP is controlled by the antagonistic action of two classes of enzymes: diguanylate cyclases (DGCs) and phosphodiesterases (PDEs). DGCs catalyze the formation of c-di-GMP from two GTP molecules via conserved GGDEF domains containing either a GGDEF or GGEEF catalytic motif, and this reaction typically requires dimerization (Paul et al. 2004). The enzymatic activity of many DGCs is subject to feedback inhibition through binding of c-di-GMP to a conserved allosteric site known as the I-site (Christen et al. 2006). In contrast, degradation of c-di-GMP is mediated by PDEs that carry either EAL or HD-GYP domains. EAL-domain PDEs hydrolyze c-di-GMP to linear pGpG (5’-phosphoguanylyl-(3’,5’)-guanosine), whereas HD-GYP domain PDEs hydrolyze c-di-GMP into two GMP molecules (Schmidt et al. 2005, Ryan et al. 2009). Once synthesized, c-di-GMP binds to a variety of effectors, including protein effectors and RNA riboswitches, that trigger downstream responses, as shown in Fig. 2A. These responses often coordinate the expression of virulence factors and surface-associated behaviors, such as the transition from motility to sessility, but also contribute to stress resistance, including tolerance to antibiotics and bacteriophages. For a more detailed overview of c-di-GMP signaling mechanisms see (Jenal and Malone 2006, Schirmer and Jenal 2009, Jenal et al. 2017, Valentini and Filloux 2019, Aline Dias da et al. 2020, Cancino-Diaz et al. 2023, Liu et al. 2025).

Genomic analyses have revealed that GGDEF, EAL and HD-GYP domains are evolutionarily conserved and widely distributed across all bacterial species. Most bacterial genomes encode multiple proteins containing these domains, often in combination with different sensory input domains. This modular organization allows c-di-GMP signaling systems to integrate a wide range of environmental and intracellular cues (Randall et al. 2022). Furthermore, a particular subset of these proteins, known as dual-domain proteins, contains both GGDEF and either EAL or HD-GYP domains within a single polypeptide. Interestingly, in some cases one or both domains are degenerate and lack the conserved amino acid residues required for enzymatic activity (Christen et al. 2005, Schmidt et al. 2005). Nevertheless, even degenerate domains may retain the ability to bind c-di-GMP. Such binding can induce conformational changes that modulate the activity of a neighboring functional domain or promote interaction with downstream effectors (Newell et al. 2009). Through these mechanisms, degenerate domains, whether present in proteins with multiple domains or alone, can still play important regulatory roles.

The specificity of c-di-GMP-mediated responses can be explained by two non-mutually exclusive models (Junkermeier and Hengge 2023), shown in Fig. 2B. In the global signaling model, also known as the Bow-tie model, c-di-GMP is produced and degraded by multiple enzymes, resulting in a cytosolic pool that is sensed by different effectors with different binding affinities (Yan et al. 2017). In contrast, the local signaling model, also known as the Hub model, assumes that specific DGCs, PDEs and effectors form spatially organized signaling complexes. These protein-protein interactions allow c-di-GMP to act in a localized manner, leading to distinct regulatory outputs. Such compartmentalization could explain why only a subset of DGCs and PDEs contribute to specific cellular phenotypes (Hengge 2021). Recent network-based analyses have provided further support for the local signaling paradigm. In particular, dual-domain proteins may function as potential central hubs within the local signaling model. These multifunctional proteins may coordinate input integration and signal transduction across different regulatory pathways (Vasenina et al. 2024).

## Significance of global c-di-GMP signaling in *Bordetella*

The first experimental evidence for c-di-GMP-dependent regulation in *Bordetella* species was provided in *B. bronchiseptica*, where Sisti *et al*. demonstrated that heterologous expression of DGC and PDE from *Pseudomonas aeruginosa* significantly altered bacterial phenotypes. Specifically, expression of a DGC enhanced biofilm formation and simultaneously reduced swimming motility, whereas PDE expression suppressed biofilm formation without affecting motility in soft agar assays (Sisti et al. 2013). These results provided the first evidence that intracellular c-di-GMP levels directly modulate *Bordetella* surface-associated behaviors.

Subsequent studies have confirmed these results. Increased intracellular c-di-GMP concentrations not only promoted biofilm development but also suppressed the expression of virulence factors, particularly the type III secretion system (T3SS), a major contributor to *B. bronchiseptica* cytotoxicity (Belhart et al. 2019, Belhart et al. 2023). To examine the global effects of c-di-GMP signaling, Gutierrez *et al*. ectopically expressed native *Bordetella* enzymes, namely the DGC BdcA (BB3576) and the PDE PdeA (BB2664), which was followed by comparative multi-omics analysis (Gutierrez et al. 2022).

Label-free quantitative proteomics identified 64 differentially abundant proteins in strains with artificially modulated c-di-GMP levels, including several virulence-associated factors such as the T3SS protein Bsp22, adenylate cyclase toxin, adhesin-processing enzymes, outer membrane proteins, and various stress-related and metabolic proteins. Parallel transcriptome profiling revealed 358 differentially expressed genes under high c-di-GMP conditions. These included upregulation of phage-related genes and stress-associated genes and downregulation of motility regulators (*flhDC*) and components of the *bcs* T3SS locus (Gutierrez et al. 2022). Functionally, strains with elevated levels of c-di-GMP exhibited reduced cytotoxicity towards eukaryotic cells *in vitro*. In mouse infection models, these strains demonstrated decreased colonization and persistence in the respiratory tract and elicited a weaker immune response compared to the wild-type strain (Belhart et al. 2019, Gutierrez et al. 2022). Overall, these findings highlight the central role of c-di-GMP signaling in shaping physiology of *B. bronchiseptica*.

## Molecular components of *Bordetella* c-di-GMP signaling

Genomic analyses reveal that classical *Bordetella* species encode multiple proteins containing GGDEF, EAL and HD-GYP domains, which are typically associated with c-di-GMP synthesis and degradation. In *B. bronchiseptica* strain RB50 (*Bb*), 21 putative c-di-GMP-metabolizing enzymes and/or their degenerate variants have been identified. These include 10 predicted DGCs, 7 PDEs and 4 dual-domain proteins that harbor both GGDEF and EAL domains. In contrast, the genome of *B. pertussis* strain Tohama I (*Bp*), which has undergone extensive reduction during evolution, encodes only 9 predicted non-mutagenized c-di-GMP-metabolizing enzymes in comparison to *B. bronchiseptica*. Of these, 8 are homologous to their counterparts in *B. bronchiseptica*, while one, BdcK (BP1492), is unique to *B. pertussis*, suggesting species-specific functional adaptation of c-di-GMP network. The genomes of ovine-adaptive *B. parapertussis*_OV_ strain Bpp5 (*Bpp*_OV_) and human-adapted *B. parapertussis*_HU_ strain 12 822 (*Bpp*_HU_) each encode 18 predicted non-mutagenized c-di-GMP-metabolizing proteins in comparison to *B. bronchiseptica*. Interestingly, neither *B. parapertussis* lineage encodes BdcK, supporting the conclusion that this enzyme is specific to *B. pertussis*.

**Figure 3.**
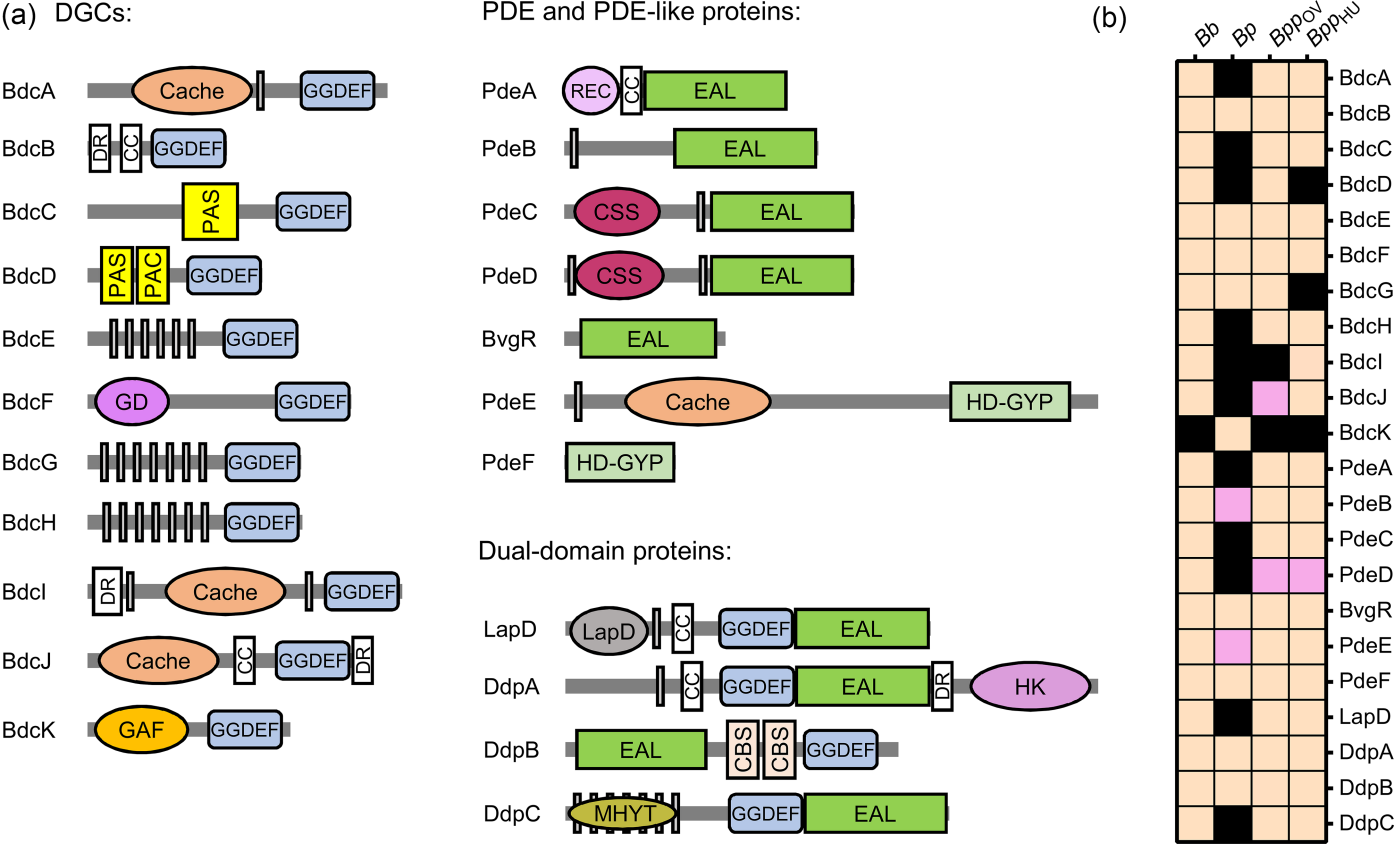
Inventory of DGCs, PDEs and dual domain proteins in classical *Bordetella* species. **(A)** Schematic representation of the domain architecture of predicted diguanylate cyclases (DGCs), phosphodiesterases (PDEs), and dual-domain proteins in classical *Bordetella* species. DGCs contain GGDEF domains, PDEs contain either EAL or HD-GYP domains, and dual-domain proteins harbor combinations of these motifs. Many proteins also include additional sensory domains, such as Cache, PAS, GAF, and REC, which are involved in signal detection and modulation of catalytic activity. GD, globin-like domain; LapD, LapD domain; HK, histidine kinase-like domain; DR, disordered region, CC, coiled-coil motif; transmembrane regions are represented with light-grey vertical bars. **(B)** Distribution of genes encoding DGCs, PDEs, and dual-domain proteins across classical *Bordetella* species. The figure shows gene presence and mutations in *B. pertussis* Tohama I (*Bp*), ovine *B. parapertussis* Bpp5 (*Bpp*_OV_), and human-adapted *B. parapertussis* 12 822 (*Bpp*_HU_) relative to *B. bronchiseptica* RB50 (*Bb*), which is used as the reference strain; the only exception is BdcK, which is specific to *B. pertussis*. Each row represents a specific protein, and each column corresponds to a strain. Color shading indicates the predicted protein status: orange for intact proteins, pink for proteins with mutations as compared to *Bb*, and black for gene absence or lack of protein production. See text for further details.

An overview of the predicted c-di-GMP-metabolizing enzymes and their domain architecture is shown in Fig. 3A, while a comparative analysis is provided in Fig. 3B. The full amino acid sequences of *Bb* proteins, and *Bp*-specific BdcK, with domains annotated using NCBI CD-Search (Marchler-Bauer et al. 2017, Lu et al. 2020, Wang et al. 2023) and catalytic motifs highlighted as conserved or degenerate, are available in Supplementary Data (Fig. 3A Supplementary Data). Corresponding locus tags and protein identifiers used for cross-species comparisons are listed in Supplementary Data (Fig. 3B Supplementary Data). A brief description of each protein is provided below.

### Diguanylate cyclases

**BdcA** (BB3576; absent in *Bp*, present in *Bpp*_OV_ and *Bpp*_HU_), is a membrane-associated DGC with validated catalytic activity. BdcA inhibits flagellar motility, promotes biofilm formation and enhances persistence in murine airway (Sisti et al. 2013, Belhart et al. 2019). The N-terminal periplasmic region of BdcA contains a Cache domain, which is one of the most abundant extracellular sensory domains of bacteria known to bind diverse small ligands, including amino acids, organic acids and quorum-sensing autoinducers (Upadhyay et al. 2016, Randall et al. 2022). The physiological ligand of BdcA remains unidentified.

**BdcB** (BB3903; present in *Bp, Bpp*_OV_ and *Bpp*_HU_) is a cytosolic DGC that also inhibits motility, promotes biofilm formation and negatively regulates T3SS genes in *B. bronchiseptica* (Belhart et al. 2023). BdcB contains an N-terminal intrinsically disordered region (IDR), which is essential for enzymatic activity and may facilitate ligand binding or protein-protein interactions necessary for dimerization, which is a prerequisite for GGDEF domain activation. Unlike many DGCs, BdcB lacks canonical sensory or phosphorelay domains and represents the first example of a GGDEF protein with an essential IDR (Belhart et al. 2023).

**BdcC** (BB3114; absent in *Bp*, present in *Bpp*_OV_ and *Bpp*_HU_) and **BdcD** (BB2790; absent in *Bp* and *Bpp*_HU_, present in *Bpp*_OV_) are cytosolic putative DGCs containing PAS and PAS-PAC domains, respectively, which commonly serve as sensory modules (Zhulin et al. 1997, Taylor and Zhulin 1999, Stuffle et al. 2021). Although their GGDEF domains are conserved, their catalytic activity is unconfirmed.

**BdcE** (BB4664; present in *Bp, Bpp*_OV_ and *Bpp*_HU_), **BdcG** (BB2626; present in *Bp* and *Bpp*_OV_, absent in *Bpp*_HU_), and **BdcH** (BB0991; absent in *Bp*, present in *Bpp*_OV_ and *Bpp*_HU_) are structurally similar transmembrane proteins characterized by 6–7 predicted transmembrane helices and conserved C-terminal GGDEF domains. These proteins vary only slightly in size (BdcE 380 aa; BdcG 385 aa; BdcH 387 aa). While they lack identifiable accessory domain, conservation of active site motifs suggest they are functional DGCs.

**BdcF** (BB1960; present in *Bp, Bpp*_OV_ and *Bpp*_HU_), also known as *Bpe*Greg for *B. pertussis* globin regulator, promotes biofilm formation (Wan et al. 2009). BdcF belongs to the globin-coupled sensor (GCS) family of DGC. It contains an N-terminal heme-binding globin domain, connected via a linker middle domain to a C-terminal GGDEF domain. Oxygen binding stimulates its DGC activity (Wan et al. 2009). BdcF is structurally and functionally similar to DosC from *Escherichia coli*, a well-characterized oxygen-sensing GCS DGC (Tuckerman et al. 2009).

**BdcI** (BB1220; absent in *Bp* and *Bpp*_OV_, present in *Bpp*_HU_) and **BdcJ** (BB2660; absent in *Bp*, mutated in *Bpp*_OV_, present in *Bpp*_HU_) are putative membrane-associated DGCs with periplasmic sensory Cache domains. Their ligands and physiological functions remain to be determined. BdcJ has a degenerate active site motif, GADEF. In *Bpp*_OV_, the *bdcJ* gene (BPP5_2486) encodes a variant with a divergent and extended C-terminal region of the GGDEF domain. The degenerate GADEF motif is retained.

**BdcK** (BP1492; present exclusively in *Bp*) is a unique cytosolic DGC present in all sequenced *B. pertussis* isolates. The *bdcK* gene in *B. pertussis* Tohama I strain is flanked by two IS481 elements, suggesting potential genomic mobility. BdcK contains an N-terminal GAF domain, which in other systems binds nucleotides or small intracellular ligands (Heikaus et al. 2009), implying that BdcK may respond to internal signals.

### Phosphodiesterases and phosphodiesterase-like proteins

**PdeA** (BB2664; absent in *Bp*, present in *Bpp*_OV_ and *Bpp*_HU_) is an active cytosolic PDE that, when overexpressed, inhibits biofilm formation in the Bvg^i^ phase (Gutierrez et al. 2022). PdeA contains an accessory N-terminal phosphoreceiver (REC) domain and a C-terminal EAL domain. REC domains are typically phosphorylated by a cognate sensory kinase (Galperin 2010, Randall et al. 2022), suggesting that PdeA may be part of a two-component signaling pathway.

**PdeB** (BB2110; mutated in *Bp*, present in *Bpp*_OV_ and *Bpp*_HU_) is a putative PDE with a conserved EAL domain but no identified accessory domains. Interestingly, only 11 residues are predicted to be localized to the periplasmic space. The *Bp* variant (BP1460) lacks the N-terminal 108 amino acids present in the *Bb* PdeB, while the C-terminal EAL domain remains intact.

**PdeC** (BB3116; absent in *Bp*, present in *Bpp*_OV_ and *Bpp*_HU_) and **PdeD** (BB3128, absent in *Bp*, mutated in *Bpp*_OV_ and *Bpp*_HU_) are homologous membrane-associated putative PDEs sharing 28% sequence identity. Both proteins contain a conserved CSS motif at their N-terminus, which includes two conserved cysteine residues. These cysteines may function as redox switches and form a disulfide bond under oxidizing conditions, thereby preventing PDE dimerization and inhibiting the enzymatic activity of the C-terminal EAL domain. For additional information on CSS motif-mediated regulation of PDEs see (Herbst et al. 2018). The *Bpp* variants of PdeD (BN117_RS23995 in *Bpp*_OV_, BPP2807 in *Bpp*_HU_) lack 198 and 16 N-terminal amino acids, respectively, while the EAL domain remains intact.

**BvgR** (BB2996; present in *Bp, Bpp*_OV_ and *Bpp*_HU_) is primarily known as a transcriptional repressor that silences *vrgs* expression during the Bvg⁺ phase (Merkel and Stibitz 1995), but recent findings suggest it plays a more nuanced regulatory role (Coutte et al. 2016, Coutte et al. 2024, Gutierrez et al. 2024, Nicholson et al. 2024). Notably, BvgR lacks a DNA-binding domain, raising questions about its mechanism of action. Based on structural homology, it has been hypothesized that BvgR may function as a PDE, modulating c-di-GMP levels to exert regulatory control (Coutte et al. 2016, Chen et al. 2017, Chen and Stibitz 2019). However, detailed sequence analyses indicate that BvgR is likely catalytically inactive, as it lacks the conserved residues required for PDE activity (Gutierrez et al. 2024). These results support an alternative model in which BvgR does not act as an active PDE, but rather as a non-catalytic c-di-GMP effector that mediates regulation through protein-protein interactions.

**PdeE** (BB1564; mutated in *Bp*, present in *Bpp*_OV_ and *Bpp*_HU_) is a putative membrane-bound PDE with a periplasmic Cache domain at its N-terminus and a cytosolic HD-GYP domain. Although its domain architecture suggests it responds to extracellular signals, catalytic activity has not yet been demonstrated. It has a degenerate active site motif, QD-GYP, instead of the canonical HD-GYP. The *Bp* variant (BP0880) lacks the C-terminal 170 amino acids present in *Bb* PdeE, which correspond to the majority of the HD-GYP domain.

**PdeF** (BB1961; present in *Bp, Bpp*_OV_ and *Bpp*_HU_) is a putative HD-GYP PDE encoded adjacent to *bdcF* (BB1960), suggesting potential functional coupling between BdcF and PdeF.

### Dual-domain proteins

**LapD** (BB1184; absent in *Bp*, present in *Bpp*_OV_ and *Bpp*_HU_) is a membrane associated dual-domain protein with degenerate GGDEF and EAL domains. It regulates the surface localization of BrtA, an RTX-family adhesin involved in biofilm formation under Bvg^-^ conditions (Ambrosis et al. 2016, Nishikawa et al. 2016). LapD does not appear to be involved in c-di-GMP-dependent motility inhibition (Belhart et al. 2019).

**DdpA** (BB2109; present in *Bp, Bpp*_OV_ and *Bpp*_HU_) is a membrane-associated dual-domain protein that lacks conserved catalytic residues in both the DGC and PDE domains. DdpA (BB2109) of *B. bronchiseptica* is required for activation of catalytic activity of BdcA. Its deletion in *B. bronchiseptica* abolishes BdcA-dependent motility inhibition and biofilm formation, indicating a functional interaction between these two proteins (Belhart et al. 2019). Interestingly, although *B. pertussis* does not encode a BdcA homolog, deletion of *B. pertussis ddpA* (*bp1092*) alters the expression of virulence factors and impairs *B. pertussis* intracellular survival in THP-1 macrophages (Debandi et al. 2024). DdpA also contains a BaeS-like sensor domain frequently found in histidine kinases (HK) (Raffa and Raivio 2002).

**DdpB** (BB2957; present in *Bp, Bpp*_OV_ and *Bpp*_HU_) is a cytosolic putative dual-domain protein that contains two CBS (cystathionine-β-synthase) domains located between its GGDEF and EAL domains. However, the catalytic motifs of enzymatic domains are mutated to GGDDF and ESL, respectively. The impact of these substitutions on enzymatic activity is unknown, and neither DGC or PDE activity has been reported. CBS domains are presumed to associate in a dimeric assembly known as a Bateman module, in which the interdomain interface forms a ligand-binding cleft. Ligand binding at this site can modulate the overall protein conformation and may influence the activity and/or interactions of DdpB (Zhang et al. 1999, Baykov et al. 2011, Ereno-Orbea et al. 2013).

**DdpC** (BB3317; absent in *Bp*, present in *Bpp*_OV_ and *Bpp*_HU_) is a membrane-associated dual-domain protein containing a MHYT domain. The MHYT domains have been proposed to function as a sensor domain capable of binding oxygen, CO or NO (Galperin et al. 2001). Both the GGDEF and EAL domains are conserved, but their enzymatic activity remains untested.

### Effectors of c-di-GMP molecules

The downstream effects of c-di-GMP signaling are mediated by specific effectors that bind this second messenger and trigger diverse cellular responses (see Fig. 2A). However, in contrast to the relatively straightforward prediction of the catalytic domains in DGCs and PDEs, the identification of c-di-GMP-binding effectors remains more challenging. Among protein effectors, one class includes those with degenerate GGDEF or EAL domains that have lost their catalytic activity but retain the ability to bind c-di-GMP. Another well-characterized class consists of proteins with PilZ domains, that serve as conserved c-di-GMP-binding modules in many bacterial species (Amikam and Galperin 2006).

The *Bordetella* genome encodes a single PilZ domain-containing protein, YcgR (BB1561; BP0877 in *Bp*, BPP5_1323 in *Bpp*_OV_, BPP2164 in *Bpp*_HU_). In *E. coli*, YcgR binds c-di-GMP and interacts with the proteins of the flagellar switch complex FliG and FliM, which act as a flagellar brake and inhibit motility (Paul et al. 2010). However, this function does not appear to be conserved in *B. bronchiseptica*. Experimental evidence indicates that deletion of *ycgR* does not affect flagellar motility, suggesting that YcgR either lacks functional activity or is involved in alternative, as yet unidentified, regulatory pathways (Belhart et al. 2019).

### Comparative insights and evolutionary perspective

The classical *Bordetella* species share a common evolutionary origin, and comparative genomic analyses indicate that *B. pertussis* and *B. parapertussis* evolved independently from a *B. bronchiseptica*-like ancestor through genome reduction (Parkhill et al. 2003, Diavatopoulos et al. 2005). This reductive evolution is reflected also in the composition of their c-di-GMP signaling network.

*B. bronchiseptica*, which persists in environmental reservoir and infects a broad range of mammals, encodes 21 predicted c-di-GMP-metabolizing proteins or their degenerate variants. Many of these proteins are linked to sensory domains, likely integrating diverse signals. In contrast, the human-adapted *B. pertussis* encodes only nine predicted non-mutagenized c-di-GMP-metabolizing proteins. This reduction is consistent with its strict host specialization and dependence on direct host-to-host transmission, and has been observed in other highly adapted bacterial pathogens (Bobrov et al. 2011, Rotcheewaphan et al. 2016). Despite its reduced c-di-GMP repertoire, *B. pertussis* retains several key components of the c-di-GMP signaling system, including the transcriptional regulator BvgR, the globin-coupled DGC, BdcF (also known as *Bpe*GReg), and the associated PDE, PdeF, as shown in Fig. 3B. It also encodes the dual-domain protein DdpA (BP1092), a homolog of BB2109 of *B. bronchiseptica* (Debandi et al. 2024). These retained components likely represent core elements of c-di-GMP signaling network essential for host interaction.

Interestingly, *B. pertussis* has also acquired a unique DGC, BdcK, which appears to be specific to this species. The functional role of BdcK remains to be determined, raising two important questions: does BdcK compensate for the reduced signaling network by taking over functions of another DGC present in *B. bronchiseptica*, or does it provide a novel function specific to *B. pertussis*? The BdcK is also absent from both *B. parapertussis* lineages, *B. parapertussis*_HU_ and *B. parapertussis*_OV_, which similarly exhibit a reduction in c-di-GMP signaling network compared to *B. bronchiseptica*. However, the reduction in these strains is less extensive than that observed in *B. pertussis*, as illustrated in Fig. 3B.

## Specific regulatory modules and phenotypic outcomes

Although the *Bordetella* genome encodes numerous proteins predicted to participate in c-di-GMP signaling, only a subset has been functionally characterized at the protein level. Below, we review the known regulatory modules and their associated phenotypic consequences.

### Regulatory function of BvgR and hypothetical mechanism of its action

BvgR is a pivotal regulatory protein in classical *Bordetella* species and plays a central role in the modulation of virulence and environmental adaptation. It was initially identified in 1995 by Merkel and Stibitz as an essential negative regulator of *vrgs* in *B. pertussis* (Merkel and Stibitz 1995). Subsequent studies demonstrated that BvgR expression is activated by phosphorylated BvgA (BvgA∼P), which binds to the *bvgR* promoter, classifying BvgR as a member of the *vag* class (Merkel et al. 2003, Merkel et al. 1998a). Functional studies in both *B. pertussis* and *B. bronchiseptica* have shown that deletion of *bvgR* impairs respiratory tract colonization and attenuates the host immune response. In *B. pertussis*, Δ*bvgR* mutants exhibit reduced ability to induce leukocytosis, a hallmark of pertussis pathogenesis (Martinez de Tejada et al. 1998, Merkel et al. 1998b). Similarly, in *B. bronchiseptica*, Δ*bvgR* strains show reduced persistence and elicit a weaker immune response relative to the wild-type strain (Gutierrez et al. 2024).

It is now established that BvgR functions as a key repressor of *vrgs*. Transcriptomic analyses of Δ*bvgR* mutants in both *B. pertussis* and *B. bronchiseptica* show a strong upregulation of *vrgs* expression (Coutte et al. 2016, Gutierrez et al. 2024, Nicholson et al. 2024). In *B. bronchiseptica*, genes that are derepressed in the absence of BvgR include those involved in flagellar biosynthesis, most notably the master regulator *flhDC*. In Δ*bvgR, flhDC* is significantly upregulated, triggering expression of downstream motility genes such as flagellin gene *flaA*, and resulting in a motile phenotype (Fernandez et al. 2005, Gutierrez et al. 2024). These observations contributed to the early recognition of BvgR as a transcriptional repressor.

RNA-seq data, however, also suggest that BvgR plays a broader and more nuanced regulatory role, acting as both a repressor and activator of genes involved in virulence and adaptation to the environment (Coutte et al. 2016, Gutierrez et al. 2024, Nicholson et al. 2024). BvgR not only represses genes involved in motility, chemotaxis, capsule biosynthesis and c-di-GMP signaling, including several DGCs and PDEs, but also positively regulates the expression of components of the T3SS as well as several adhesins such as *prn, fimX, fim1* and *bipA*. The dysregulation of these factors in Δ*bvgR* mutants likely accounts for their attenuated virulence phenotype in murine infection models (Gutierrez et al. 2024, Nicholson et al. 2024).

Despite its central regulatory role, the molecular mechanism by which BvgR controls gene expression remains poorly understood, as illustrated in Fig. 1. Importantly, BvgR lacks any identifiable DNA-binding domain, making it unlikely that it acts as a direct transcriptional regulator. Instead, its structural homology to the EAL domains of PDEs has led to the hypothesis that BvgR may exhibit c-di-GMP hydrolytic activity, thereby modulating intracellular c-di-GMP levels and indirectly affecting the activity of downstream regulators such as RisA (Coutte et al. 2016, Chen et al. 2017, Chen and Stibitz 2019). This model is supported by the observation that BvgR antagonizes RisA, a DNA-binding response regulator, whose function was hypothesized to be modulated by c-di-GMP binding (Coutte et al. 2016). However, sequence analysis reveals that BvgR lacks several conserved residues essential for enzymatic activity, making its role as a functional PDE unlikely (Gutierrez et al. 2024). Nevertheless, BvgR retains putative c-di-GMP binding motifs, suggesting that it may act as a non-catalytic c-di-GMP effector. In this model, BvgR would modulate cellular signaling through protein-protein interactions rather than through direct hydrolysis of c-di-GMP. Such a mechanism aligns with emerging concepts of c-di-GMP signaling, in which non-enzymatic effectors translate localized c-di-GMP concentrations into specific phenotypic outcomes.

Taken together, these results identify BvgR as a versatile regulatory protein that may integrate c-di-GMP signaling to the control of virulence gene expression in *Bordetella* species. Further work is needed to elucidate the molecular details of BvgR function, particularly its potential role as a c-di-GMP-binding effector, and to determine how it interacts with the RisAS/K and other signaling pathways. Understanding these interactions and the environmental signals to which they respond, remains an important key objective for future investigations.

### Scaffold-assisted activation of BdcA diguanylate cyclase by DdpA

**Figure 4.**
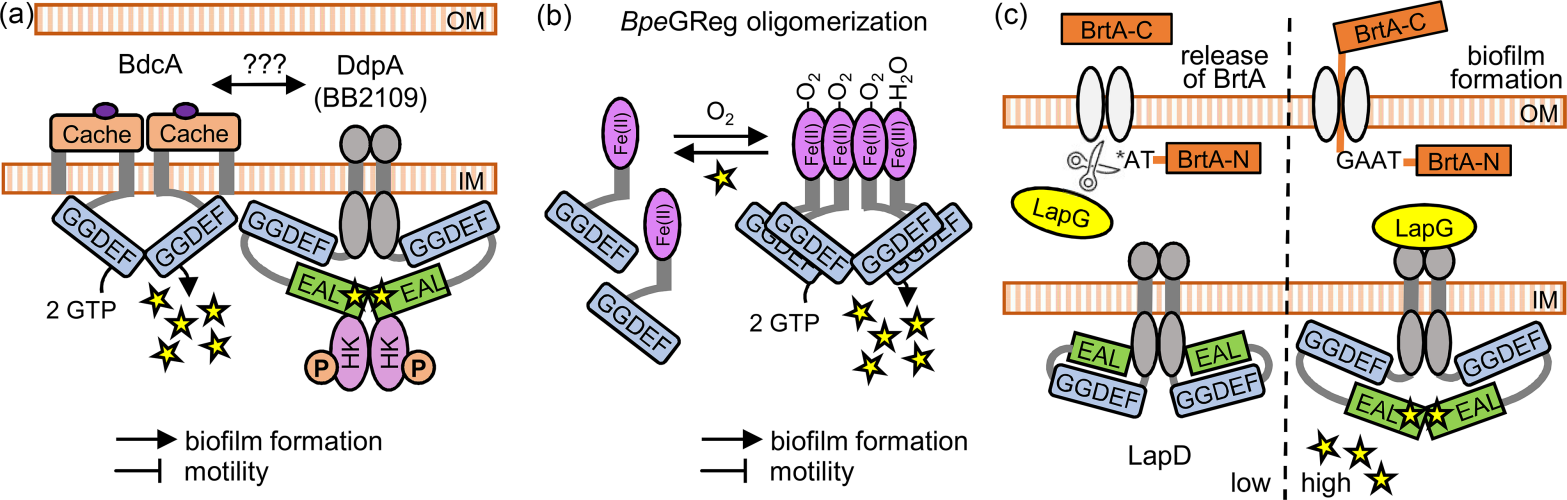
Specific regulatory modules and phenotypic outcomes. **(A)** Scaffold-assisted activation of BdcA diguanylate cyclase by DdpA. BdcA is a membrane-associated DGC with a Cache sensory domain, whose enzymatic activity depends on interaction with the dual-domain protein DdpA (BB2109). This interaction is essential for BdcA-mediated inhibition of motility and promotion of biofilm formation in *B. bronchiseptica*. **(B)** Oxygen sensing via globin-coupled diguanylate cyclase *Bpe*GReg (BdcF). *Bpe*GReg contains an N-terminal globin domain with a heme cofactor that binds oxygen and a C-terminal GGDEF domain responsible for c-di-GMP synthesis. Both oxygen binding to the heme iron in the globin domain and c-di-GMP binding to I-site in the cyclase domain regulate the oligomerization state of *Bpe*Greg. The oxygen-bound tetramer represents the most active form of the enzyme. **(C)** BrtA–LapD–LapG regulatory axis controlling surface adhesion and biofilm formation. In *B. bronchiseptica*, c-di-GMP binding to LapD sequesters the periplasmic protease LapG, preventing cleavage and release of the surface adhesin BrtA. This mechanism promotes BrtA retention at the cell surface and facilitates adhesion and biofilm formation under Bvg⁻ conditions. BrtA-C, BrtA C-terminus; BrtA-N, BrtA N-terminus; OM, outer membrane; IM, inner membrane.

Among the DGCs encoded in the genome of *B. bronchiseptica*, BdcA (BB3576) is one of the most extensively characterized. BdcA is a membrane-associated DGC composed of an N-terminal Cache sensor domain and a C-terminal GGDEF catalytic domain. Its activity has been experimentally validated through phenotypic assays and direct measurements of intracellular c-di-GMP levels (Sisti et al. 2013, Belhart et al. 2019). Overexpression of BdcA in *B. bronchiseptica* promotes biofilm formation and inhibits flagellar motility, while deletion of the *bdcA* gene results in increased motility and reduced c-di-GMP levels. However, biofilm formation on abiotic surfaces is not impaired in the Δ*bdcA* mutant, suggesting that BdcA primarily acts as a repressor of motility and that other c-di-GMP pathways contribute to biofilm development. The BdcA is absent in *B. pertussis*. Importantly, the Δ*bdcA B. bronchiseptica* strain exhibits reduced persistence in the mouse respiratory tract compared to the wild-type strain, underscoring the relevance of BdcA-mediated c-di-GMP signaling in host colonization (Belhart et al. 2019). Although the specific ligand sensed by the Cache domain of BdcA has not been identified, analogous domains in other bacteria bind small molecules such as amino acids, organic acids and quorum-sensing autoinducers (Randall et al. 2022). The catalytic activity of BdcA and its downstream effects in *B. bronchiseptica* require the presence of DdpA (BB2109), a membrane-associated protein containing degenerate GGDEF and EAL domains, along with a predicted histidine kinase domain (Fig. 4A). Deletion of *ddpA* abolishes the BdcA-dependent inhibition of motility and stimulation of biofilm formation, indicating a functional interaction between the two proteins. This interaction appears to be specific, as deletion of another dual-domain protein, LapD, has no effect on BdcA activity or regulation of motility (Belhart et al. 2019). The BdcA-DdpA relationship resembles the regulatory mechanism described for the GcbC-LapD system in *P. fluorescens*, where GcbC, a Cache domain DGC, requires interaction with the dual-domain protein LapD to catalyze c-di-GMP production. In that system, the addition of citrate, a known ligand of the Cache domain of GcbC, enhances GcbC-LapD interaction and stimulates enzymatic activity (Giacalone et al. 2018). Whether BdcA in *B. bronchiseptica* is similarly regulated by a ligand-dependent mechanism remains to be determined. Moreover, how the BdcA-DdpA integrates into the broader c-di-GMP signaling network of *B. bronchiseptica* is also unanswered.

Interestingly, although *B. pertussis* lacks a *bdcA* gene, it encodes BP1092, an ortholog of *B. bronchiseptica* DdpA, which is upregulated during macrophage infection (Lamberti et al. 2016). The precise function of *B. pertussis* DdpA is unclear, but deletion of *B. pertussis ddpA* alters the expression of key virulence genes, including *cyaA* and *fhaB*, and impairs intracellular survival in macrophages (Debandi et al. 2024). Whether *B. pertussis* DdpA interacts with one of the remaining DGCs encoded by this species remains to be determined. Nevertheless, it is tempting to speculate that DdpA proteins in both *B. bronchiseptica* and *B. pertussis* function as regulatory adaptor proteins within spatially localized c-di-GMP signaling circuits, analogous to dual-domain scaffolds described in other bacterial systems (Newell et al. 2009, Cooley et al. 2016, Giacalone et al. 2018, Christensen et al. 2020, Poli et al. 2024).

### Oxygen sensing through a globin-coupled diguanylate cyclase

An important adaptation that facilitates long-term bacterial persistence in the host is the formation of biofilms, which provide protection against host immune responses and antibiotic treatment. Biofilm-like structures have been observed *in vivo* on the surface of ciliated respiratory epithelial cells in the mammalian respiratory tract during *B. pertussis* and *B. bronchiseptica* infections (Sloan et al. 2007, Conover et al. 2010, Fullen et al. 2023). One regulator of biofilm formation in *B. pertussis*, at least *in vitro*, is an oxygen-responsive DGC termed BdcF, but also known as *Bpe*GReg (for *B. pertussis* globin regulator, BP3507) (Wan et al. 2009).

*Bpe*GReg is a member of the globin-coupled sensor (GCS) family (Freitas et al. 2003) and is conserved in the genomes of classical *Bordetella* species. It consists of an N-terminal globin domain coordinating a heme cofactor, a C-terminal GGDEF domain responsible for c-di-GMP synthesis, and a middle linker domain connecting the two (Wan et al. 2009, Wan et al. 2017). It has been reported that oxygen binding to the heme iron promotes oligomerization of the GGDEF domains and activation of *Bpe*GReg. The full enzymatic activity depends on this oligomerization, with the oxygen-bound tetrameric assembly being the most active form (Wan et al. 2009, Burns et al. 2014, Burns et al. 2016). Interestingly, in this tetramer, as shown in Fig. 4B, both monomers of one dimer are in the Fe(II)-O_2_ state, while the other dimer contains one Fe(III)-H_2_O and one Fe(II)-O_2_ state (Rivera et al. 2018). In addition to oxygen-dependent activation, *Bpe*GReg is also subject to feedback inhibition through the binding of c-di-GMP to the I-site, which provides autoregulatory control by shifting the equilibrium away from the tetrameric form (Wan et al. 2009, Burns et al. 2016).

The dissociation constant of *Bpe*GReg for O_2_ is low (Kd = 0.17 μM), suggesting that *Bpe*GReg is likely to be saturated with oxygen under physiological conditions and possibly even under the microaerobic conditions encountered during host colonization (Germani et al. 2020). Nevertheless, the *Bpe*GReg might respond to subtle changes in intracellular oxygen levels associated with metabolic changes. When *Bpe*GREg is heterologously expressed in *Salmonella* Typhimurium, it enhances extracellular polysaccharide (EPS) production, stimulates biofilm formation and inhibits flagellar motility, consistent with its function as an active DGC. In addition, a *B. pertussis* strain lacking *Bpe*GReg shows significantly reduced biofilm formation *in vitro*, supporting its role in modulating surface-associated behavior (Wan et al. 2009).

*Bpe*GReg shares amino acid similarity with DosC (Direct oxygen sensing Cyclase), the prototypical oxygen-sensing DGC in *E. coli*. In *E. coli*, DosC is co-expressed with the PDE DosP, which contains an N-terminal heme-bound O_2_-sensing PAS domain and a C-terminal EAL domain (Tuckerman et al. 2009, Shimizu 2013). Together, DosC and DosP form part of a regulatory complex known as the oxydegradosome, which links c-di-GMP signaling to RNA degradation and processing (Tuckerman et al. 2011, Gilles-Gonzalez and Sousa 2019). Although *B. pertussis* does not have a DosP homolog, the gene adjacent to *bpeGReg* encodes a predicted HD-GYP-type PDE, PdeF (BP3508). Whether *Bpe*GReg and PdeF functionally interact and/or play a similar role in RNA metabolism as DosC and DosP is unknown. Interestingly, *Bpe*GReg can inhibit motility even when its GGDEF domain is deleted, suggesting it exerts c-di-GMP-independent regulatory effects, possibly through direct protein–protein interactions (Wan et al. 2017).

Collectively, these results identify *Bpe*GReg as a globin-coupled oxygen sensor that modulates the behavior of *B. pertussis* in response to oxygen availability. However, its contribution to pathogenesis in infection models has not yet been characterized, and the downstream pathways linking c-di-GMP signaling to biofilm development remain to be elucidated. Nevertheless, DGCs such as *Bpe*GReg illustrate how classical *Bordetella* species could integrate environmental cues into regulatory networks that may enhance virulence.

### Protease control of BrtA localization through c-di-GMP signaling

In *Bordetella* species, the only c-di-GMP-dependent regulatory mechanism known to directly affect biofilm formation is the surface localization of BrtA, a member of the RTX-family of adhesins. This pathway is conserved in *B. bronchiseptica* and *B. parapertussis* but absent in *B. pertussis*. Its molecular mechanism has been inferred from studies in *P. fluorescens*, where the homologous adhesin LapA plays a critical role in surface attachment and early biofilm development (Hinsa et al. 2003).

BrtA was initially identified by Nishikawa *et al*. in the genome of *B. bronchiseptica* (Nishikawa et al. 2016). It is a high molecular weight, surface-exposed protein whose expression is repressed by BvgR in the virulent Bvg^+^ phase and upregulated under Bvg^-^ conditions. Interestingly, BrtA is also expressed during *in vivo* infection of rat tracheal epithelium (Nishikawa et al. 2016). Deletion of *brtA* gene reduces adhesion to abiotic surfaces and impairs biofilm formation under Bvg⁻ conditions, however, no significant differences in colonization have been observed between wild type and mutant *B. bronchiseptica* strains in mouse or rat infection models (Ambrosis et al. 2016, Nishikawa et al. 2016). These observations suggest that BrtA mainly contributes to adhesion to abiotic surfaces and *ex vivo* biofilm formation.

Structurally, BrtA protein contains N-terminal retention module, repeat region composed of CADG (dystroglycan-type cadherin-like) and VCBS (*Vibrio, Colwellia, Bradyrhizobium*, and *Shewanella*) repeats, a von Willebrand factor type A (vWFA) domain, an RTX repeat region, and a C-terminal signal for secretion via the type 1 secretion system (T1SS) (Nishikawa et al. 2016). Interestingly, BrtA repeat architecture differs from homologous adhesins such as LapA of *P. fluorescens* or RtxA of *Legionella pneumophila* (Smith et al. 2018). Moreover, the number of CADG/VCBS repeats varies among *B. bronchiseptica* strains, ranging from 2 to 15 repeats (with an average of 3), and appears independent of isolate origin. The *brtA* gene is co-transcribed with *lapD* and *lapG*, whose products are thought to function analogously to LapD and LapG of *P. fluorescens* (Ambrosis et al. 2016).

In *P. fluorescens*, LapD and LapG regulate the surface retention of LapA in response to intracellular c-di-GMP levels (Newell et al. 2009, Newell et al. 2011). Under conditions of low c-di-GMP, LapD remains in an autoinhibited conformation due to the absence of c-di-GMP binding to its degenerate EAL domain. In this state, LapD cannot sequester the periplasmic protease LapG, which is thus free to cleave LapA at a conserved Ala-Ala motif within the N-terminal retention module, leading to its release from the cell surface and inhibition of surface attachment. By contrast, at high c-di-GMP levels, c-di-GMP binding induces a conformational change in LapD that extends its periplasmic domain and allows sequestration of LapG. As a result, LapG is no longer able to process LapA, which remains anchored at the cell surface, promoting stable attachment and biofilm formation (Navarro et al. 2011, Newell et al. 2011).

A similar regulatory mechanism, illustrated in Fig. 4C, appears to operate in *B. bronchiseptica*. Recombinant LapG has been shown to cleave the N-terminal region of BrtA *in vitro*. Consistent with this activity, deletion of the *lapG* gene enhances biofilm formation, whereas overexpression reduces it, supporting role of LapG as a negative regulator (Ambrosis et al. 2016). Moreover, albumin and Ca^2+^ ions have been reported to both lower intracellular c-di-GMP levels and promote BrtA processing (Mugni et al. 2025). These results suggest a functional conservation of the LapD-LapG-BrtA regulatory axis in *B. bronchiseptica*. In *P. fluorescens*, LapG is a Ca^2+^-dependent protease, and Ca^2+^ availability modulates LapA release and biofilm formation (Boyd et al. 2012), indicating an additional level of calcium-dependent regulation. Interestingly, deletion of the protease *B. bronchiseptica* LapG, which increases BrtA surface retention, results in a modest but statistically significant enhancement of *B. bronchiseptica* survival in the mouse lung 14 days post-infection (Ambrosis et al. 2016).

Overall, the LapD-LapG system represents an example of direct c-di-GMP-dependent regulation of biofilm formation in *B. bronchiseptica*. However, it is likely that additional c-di-GMP-responsive pathways also contribute to biofilm development at high intracellular c-di-GMP levels. Future studies will be needed to identify and characterize these complementary regulatory circuits.

### c-di-GMP-mediated control of T3SS injectisome

The T3SS injectisome is a complex molecular device used by many Gram-negative pathogens to translocate effector proteins directly from the bacterial cytosol into the host cytoplasm, leading to manipulation of host cell processes to the benefit of the bacterium (Troisfontaines and Cornelis 2005, Galan et al. 2014). In classical *Bordetella* species, the T3SS contributes significantly to virulence, although its expression and functionality vary among species. In *B. bronchiseptica*, the T3SS plays a well-documented role in modulating host immune responses. Effector delivery via the injectisome suppresses anti-*Bordetella* antibody production, reduces IFN-γ and increases IL-10 levels, thus promoting immune evasion and persistent colonization (Yuk et al. 2000, Skinner et al. 2005, Pilione and Harvill 2006, Nicholson et al. 2014). In *B. pertussis*, the T3SS locus remains genetically intact and encodes functional proteins. However, the role of T3SS in the pathogenesis of *B. pertussis* is less clearly defined (Fennelly et al. 2008, Kamanova 2020). To date, only two T3SS-injected proteins, BteA and BopN, have been identified in both *B. bronchiseptica* and *B. pertussis*. BteA, also known as BopC, is a cytotoxic effector that disrupts host calcium homeostasis and triggers rapid necrotic cell death (Stockbauer et al. 2003, Panina et al. 2005, Kuwae et al. 2006, Zmuda et al. 2024). BopN acts as a gatekeeper that prevents premature secretion of BteA before host cell contact, although its own effector activity remains less well characterized (Nagamatsu et al. 2009, Abe et al. 2017, Navarrete et al. 2023).

**Figure 5.**
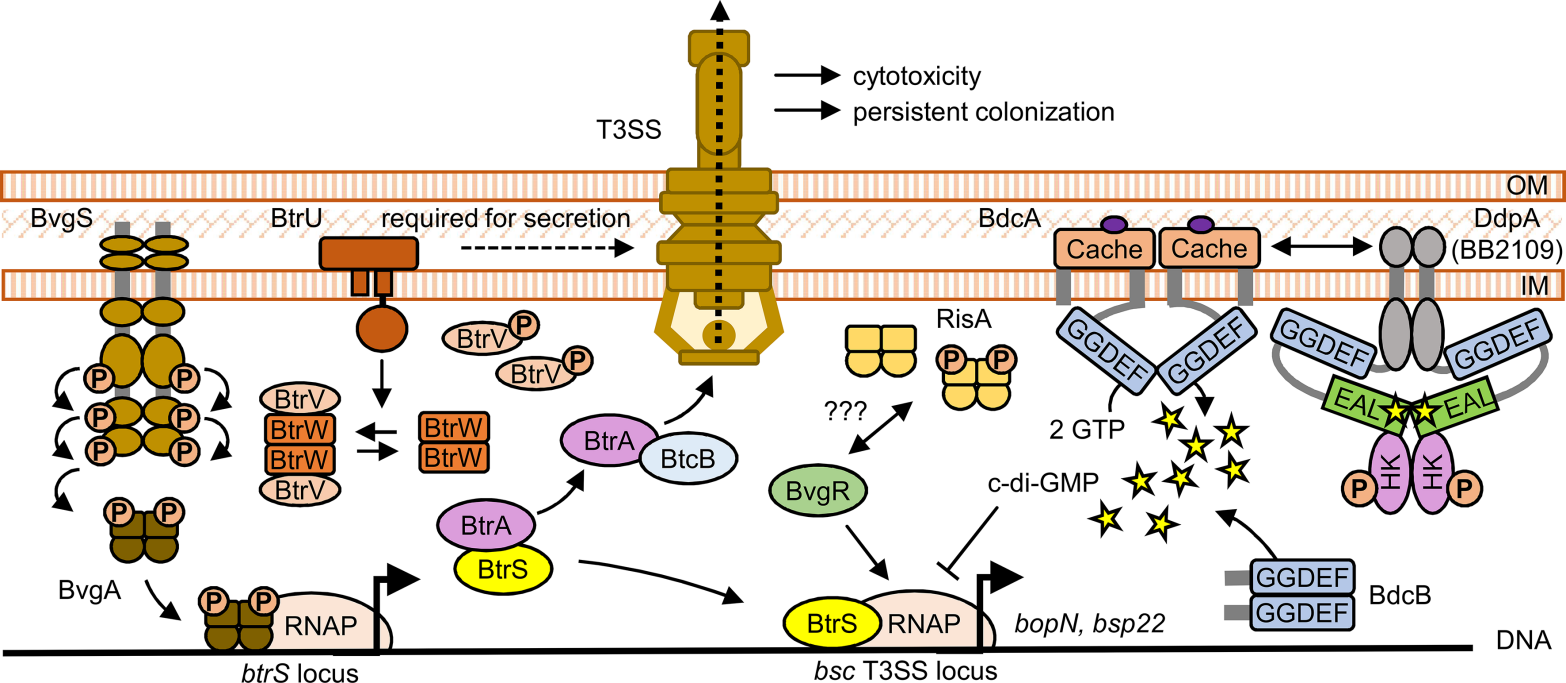
Scheme of T3SS regulation in *Bordetella* species. The expression of T3SS is primarily regulated by the BvgAS two-component system. The response regulator BvgA activates expression of the extracytoplasmic function (ECF) sigma factor BtrS, which in turn induces expression of genes in the *bsc* locus that encode structural components of the T3SS injectisome. BtrS activity is negatively regulated by the anti-sigma factor BtrA, which is secreted through the T3SS itself. Partner-switcher proteins BtrU, BtrW, and BtrV further modulate T3SS protein secretion through a phosphorylation-dependent mechanism. In addition, the DGC BdcA and BdcB act as negative regulators of T3SS gene expression. High intracellular c-di-GMP concentrations suppress transcription of both the BteA effector and structural components of the injectisome. BvgR contributes to this regulatory network by promoting T3SS gene expression, potentially through specific c-di-GMP-dependent protein-protein interactions. OM, outer membrane; IM, inner membrane.

The structural and chaperone components of T3SS are encoded by the *bsc* locus (Kerr et al. 1999, Fauconnier et al. 2001) and are under the control of the BvgAS two-component system (Yuk et al. 1998). Additional layers of transcriptional and post-transcriptional regulation involve the ECF sigma factor BtrS (BrpL), anti-sigma factor BtrA (BspR) (Kurushima et al. 2012, Ahuja et al. 2016, Drzmisek et al. 2023), the partner-switcher proteins BtrU, BtrV, BtrW (encoded by the adjacent *btr* locus) (Mattoo et al. 2004, Kozak et al. 2005), as summarized in Fig. 5, as well as the RNA-binding protein Hfq (Bibova et al. 2015, Dienstbier et al. 2019).

In addition, recent studies have shown a novel regulatory level, where high intracellular c-di-GMP concentration negatively regulates T3SS gene expression and function in *B. bronchiseptica*. Gutierrez *et al*. demonstrated that overproduction of the DGC BdcA led to transcriptional downregulation of several T3SS injectisome genes, including *bopN* and *bsp22*. This reduction in gene expression was associated with decreased macrophage cytotoxicity and reduced pro-inflammatory cytokine responses *in vivo* (Gutierrez et al. 2022). Similarly, Belhart *et al*. identified another DGC, BdcB, as a negative regulator of T3SS expression. In the absence of the *bdcB* gene, the expression of *bopB, bopD, bopN, bsp22* and *bteA* was significantly upregulated, leading to increased cytotoxicity and stronger induction of both pro-inflammatory (TNF-α, IL-6, MCP-1) and anti-inflammatory (IL-10) cytokines during macrophage infection (Belhart et al. 2023). Intriguingly, the expression of known T3SS regulators BtrS and BtrA was not affected (Belhart et al. 2023), suggesting that c-di-GMP acts downstream of or independently of this regulatory cascade, as shown in Fig. 5. Although the precise mechanisms by which c-di-GMP represses T3SS gene expression remain to be elucidated, it is plausible that this occurs through transcriptional regulation and/or modulation of transcript stability via c-di-GMP-binding proteins or RNAs. In this context, it is important to point out that BvgR, discussed above, has also been shown to positively influence T3SS expression (Gutierrez et al. 2024).

Taken together, these observations establish c-di-GMP as a potent repressor of T3SS expression and cytotoxicity towards mammalian cells. Uncovering the mechanistic basis of this repression remains an important goal for future research.

## Concluding remarks and future directions

In recent years, significant progress has been made in understanding the components and physiological roles of c-di-GMP signaling in classical *Bordetella* species. Functional studies, particularly in *B. bronchiseptica*, have shown that several DGCs, PDEs and dual-domain proteins play an important role in fine-tuning *Bordetella* surface-associated behavior and virulence. Increased intracellular c-di-GMP levels promote biofilm formation, while decreased levels enhance motility and acute virulence, which is consistent with c-di-GMP-mediated regulation in other Gram-negative bacteria. Both *in vitro* models and infection studies have shown that disruption of c-di-GMP homeostasis can alter *Bordetella* pathogenicity. However, it is important to emphasize that c-di-GMP does not function as a global on-off switch, but rather as an integrative signaling network consisting of localized regulatory modules. This modularity is illustrated by functionally coupled protein pairs, including BdcA–DdpA and LapD–LapG.

Several areas of investigation are of particular interest for future research. First, a systematic analysis of c-di-GMP-related genes in *B. pertussis, B. parapertussis* and *B. bronchiseptica* is needed to uncover species-specific signaling modules and identify the minimal essential components of this network. Second, uncovering the physiological ligands of the sensory domains and characterizing c-di-GMP effector complexes will be critical for deciphering the underlying signaling pathways. Indeed, c-di-GMP does not solely act by modulating gene expression; it can also regulate protein activity through allosteric mechanisms. This enables bacteria to rapidly respond to external cues, which may be relevant during the early stages of host colonization. Given the diversity of sensory domains found in *Bordetella* DGCs and PDEs, this network likely responds to a broad range of intracellular and environmental cues. To date, oxygen is the only direct ligand for a *Bordetella* DGC, *Bpe*GReg (BdcF) (Wan et al. 2009), but other *in vivo* relevant signals may play an equally important role. Indeed, studies have shown that host factors can modulate c-di-GMP signaling. Albumin and calcium, two molecules present in respiratory secretions, were recently shown to reduce intracellular c-di-GMP levels in *B. bronchiseptica* and inhibit biofilm formation, consistent with known role of elevated c-di-GMP in promoting biofilm formation. This effect is at least partially mediated through the LapD-LapG-BrtA pathway and can be reversed by overexpression of the DGCs BdcA or BdcB, or by simultaneous deletion of multiple PDEs and PDE-like proteins (*bvgR, pdeC, pdeD* and *pdeA*) (Mugni et al. 2025). Similarly, other factors such as iron, glutamate, ascorbic acid and/or redox signaling, which have previously been linked to T3SS regulation (Brickman et al. 2011, Hanawa et al. 2016, Goto et al. 2020), may exert their effects via c-di-GMP signaling, a hypothesis that warrants further investigation. Third, possible interactions between c-di-GMP and other nucleotide second messengers, such as c-di-AMP or (p)ppGpp are still largely unexplored and require further investigation. Fourth, elucidating the mechanism of action of BvgR, a key regulatory protein, remains an important priority.

In summary, c-di-GMP signaling is a critical, but still incompletely understood, regulatory layer in the pathophysiology of classical *Bordetella* species. Elucidating the sensory ligands and effector mechanisms of this signaling network will provide new insights into how classical *Bordetella* species adapt to and persist within host environments. Such knowledge may ultimately guide the development of new therapeutic or preventive strategies targeting these bacterial pathogens.

## Supplementary Material

fuaf065_Supplemental_Files
