# Supplementary material for: Architecture and regulatory functions of c-di-GMP signaling in classical Bordetella species
Source: FEMS Microbiol Rev. 2025 Dec 26;50:fuaf065. doi: 10.1093/femsre/fuaf065 (PMC12805831; doi:10.1093/femsre/fuaf065)

Figure 3A Supplementary Data: The full amino acid sequences of predicted c-di-GMP-metabolizing enzymes in *Bb* RB50. The BdcK sequence of *Bp* TohamaI is also provided. Sequences were analyzed by NCBI CD-Search tool (Marchler-Bauer et al. 2017, Lu et al. 2020, Wang et al. 2023)

**BdcA (****Bb RB50 AYT36_RS18170, BB3576, WP_003813543.1, 540 aa)**

dCache_1 domain, gray; GGDEF domain, green; conserved active site motif, yellow

MMRQLDLRRLILGLSMLSLLLALAGTLYASAVVQRDILLSSTLEDNRVYARKLALAADDLLANAMRDLAYGAQKIGERPDSRVIAQEVERLQAQTNRFNAVTYVDANGVFRAVAPHAGNIVGRPVVSAEMRQALEHRGALISDPFIAPTGRWLIAMSQPVFGPDGAFAGVLSGLFYLHEGNALKSLLGERDHRNGSYQYAVDRQGVLLYHPQATRIGESQTSNPLLAEVRAGRGGAQRFVNTLGTEMLAGYAVVPATGWGVVAQRPVSSVVAATQLLVRRTVYALAIWVALGVLCVWWLADRISRPLSQLARAAAELDNPRAADGALSVRAWYREALQIKNALMAVRRKIGTKFEQLRQDSLTDPLTGLLNRRGLEEYLRNTPWQDGAMTVLALDIDRFKIINDTHGHAAGDEVIQSLAGILRSSARTKDVLARL**GGEEF**AVLLPDVDVPQAPQLAERIRRSIEAAPTEAGVSYTVSIGVACYPAHGASLEAVMRCADEALYAAKAGGRNRTHLYRVGMPLPASLTDAAAGSEPSQDLRS*


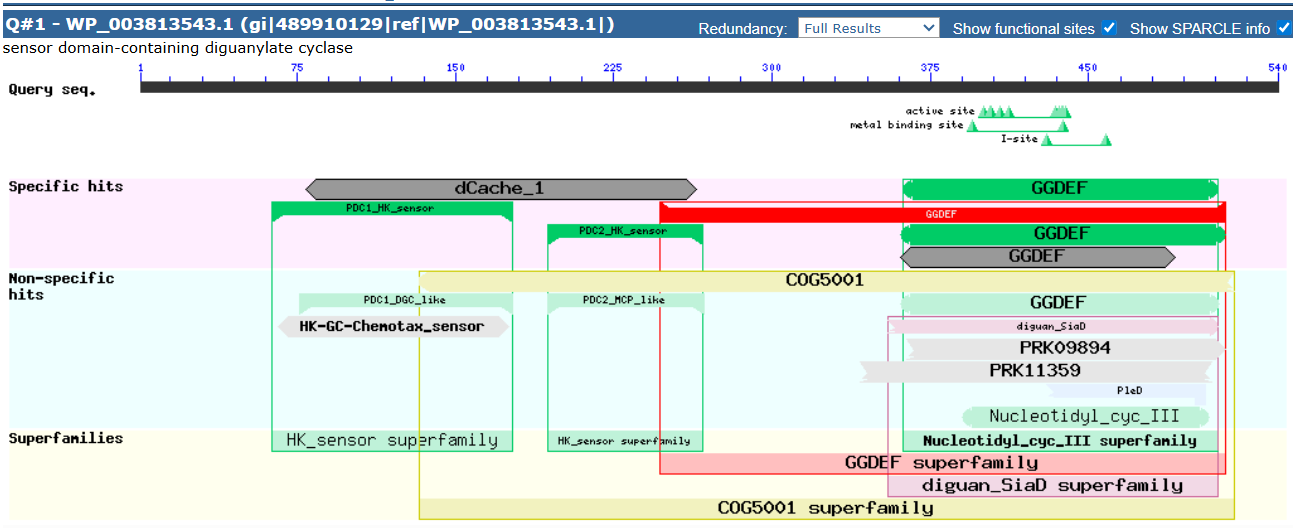


BdcB **(Bb RB50 AYT36_RS19820, BB3903, WP_003814021.1, 251 aa)**

GGDEF domain, green; conserved active site motif, yellow

MARLDNADATSMTGRPPLIMNDQRKSSRASCRASANYLRAEAALPRRRYPQPPVSGAATLQELQAQVDTLELENARLRRLALTDDLTGAYNRRYFATMLRDALRERVRGGGLALCLFDIDNFKTINDRHGHFAG**D**YLLRRVALAARRCMR**R**TS**D**DLCRV**GGDEF**AAVLSAPSASAALAQAQRVLDAIRAIAPLDTPHGPRHVTATFGLAWIAPGVSLTWEQAYSDADRALYRAKQAGKNRLHLIASRTAGA*


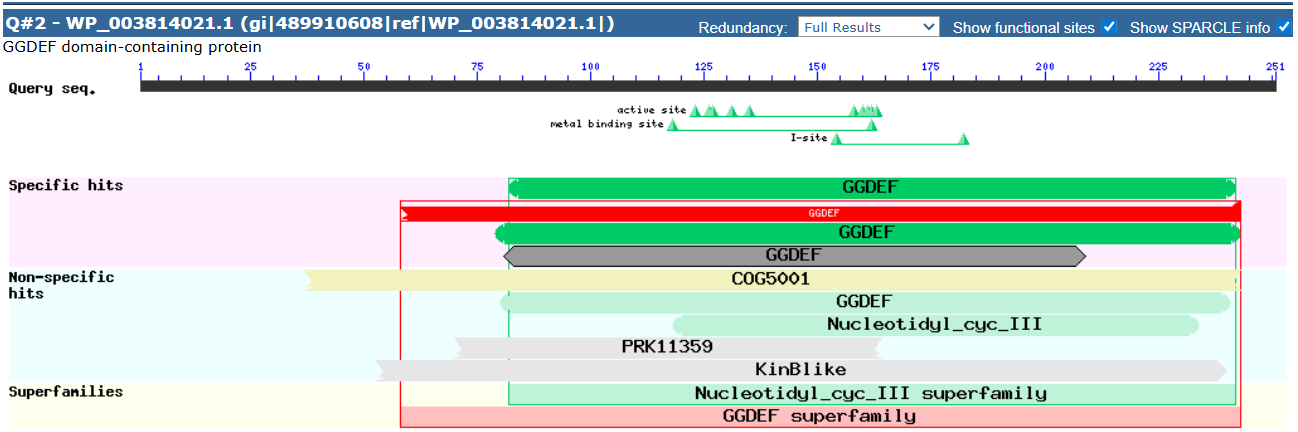


**BdcC (Bb RB50 AYT36_RS15820, BB3114, WP_230594877.1, 445 aa)**

PAS domain, gray; GGDEF domain, green; conserved active site motif, yellow

MAISPGRDFLDELVNRAVQRLGVDYAWVVLHGAKGEHARTVLSANCRMRWNGAESYLATGQSALAGVLSHATQYVCARDLERHVPGDLALRRLGAQACVAHALRDESGLQRGHVAFVFSEPLADPAIYVSALSACATFTERAVLALDAERKARQEALQLASRYQALFEKAPVLINAFDARGKCILWNLECERKFGWSVEEVNNHPEPLALFYPDPQTRARVVASVSQAPGRAFEEWHPVTRAGKVLSTLWSNIVLESGTVVNIGLDITERKRAELALTRLATIDSLTDCWNRAEILKILRQEITRLRQHPDQPLAVVMLDLDHFKQLNDLHGHLVGDAALRYFSDQLRSNLRGTDFVGRI**GGEEF**LALLPGCDGDAALAMADRLRAALRNDPFTTEGQAAPLSVSVGIAFATPEDTAVSDVMRRADTALYEAKRRGRDRAVVYFG*


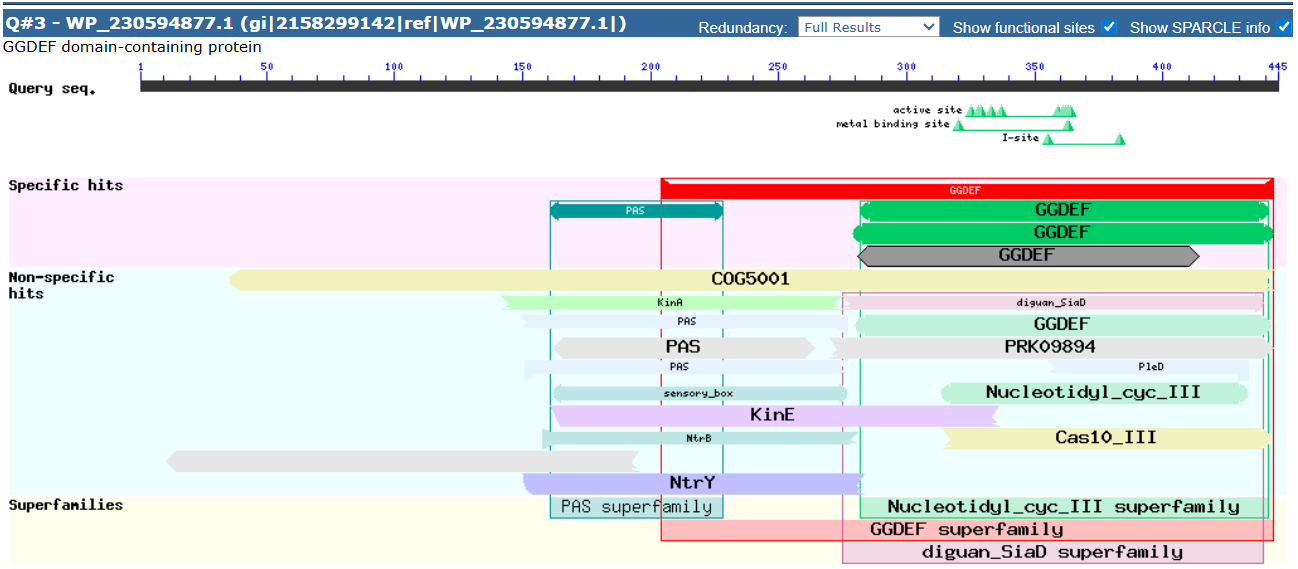


**BdcD (Bb RB50 AYT36_RS14195, BB2790, WP_003811448.1, 315 aa)**

PAS-PAC domain, gray; GGDEF domain, green; conserved active site motif, yellow

MPDASTLFNDSAVYRTLLESTKAIPWKIDWATMKFTYIGPQIEALLGWSADSWVSVEDWAMRMHPEDREYVVNYCVSQSQAGQDHEADYRALTKDNGYVWIRDVVHVVRNDKGEAEALIGFMFDITERKKTEEKLLLLQKELEVLSFKDGLTNIANRRRFDSSFDLEWERARHERQPLSMLLFDVDYFKQYNDLYGHTQGDECLVEIAQTLSLALDGPRDLVARY**GGEEF**VVLLPEADAEVARKVAERCQRLIEKKSIVHALSPHGRRVTVSIGAGTVVPGGQTAPAGFIKAVDQQLYAAKKNGRHRIEHVRLET*


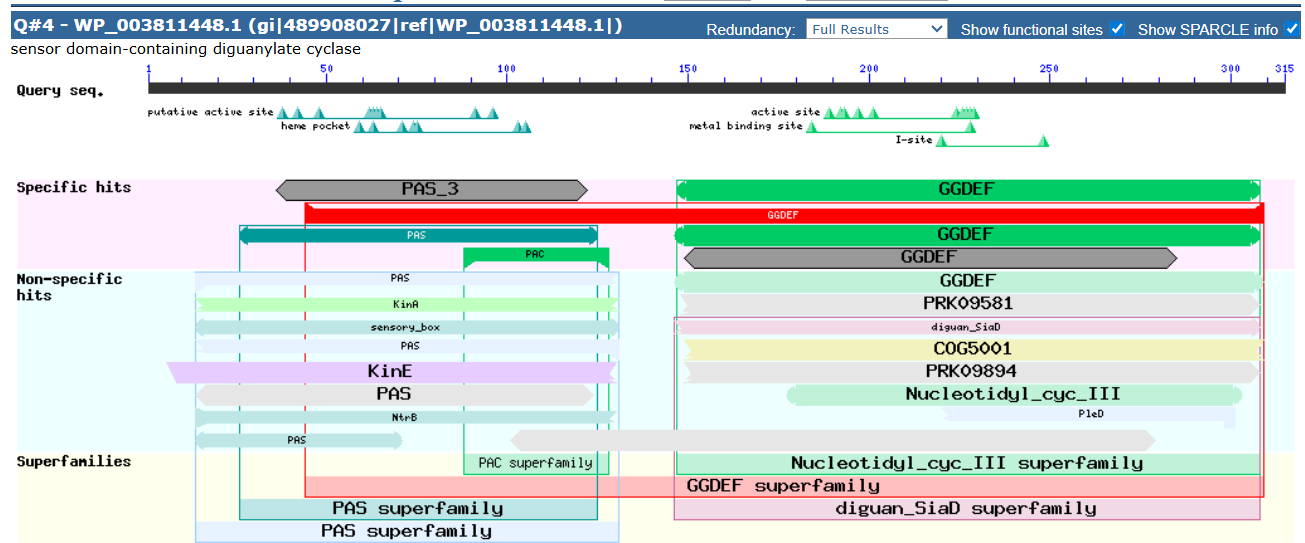


**BdcE (Bb RB50 AYT36_RS23695, BB4664, WP_010927160.1, 380 aa)**

GGDEF domain, green; conserved active site motif, yellow

MYRTDRLYSPPAWPVTRWLADPGRNVPDDIRQALLAGLFGTISIFVGGVLNSVLVALIIAIRLPQAPFIAWLAFELACCLARAVVLVSARRAAAAGRPTHTDAYIVLTVLWACSVGYGTLISVASGDWVAAILACLSAAAMVGGICFRNFSAPRLTALMICVSLGPCTTLPWFTGEHSLWIVSVQLPLYLASMTVAAYRFNRMLVATMQAERDSDRLARCDALTGLLNRFGLGFALERAVAATRRDGNEFALLYLDLDGFKSVNDTHGHAAGDRLLRDVAARLTELAPADAAIARI**GGDEF**VLLVRDCDEACATALGDRIVARICEPYDLGTPRPVRIGGSVGIALVPRHGQEMTAILKAADRALYLAKSAGKSRTALAA*


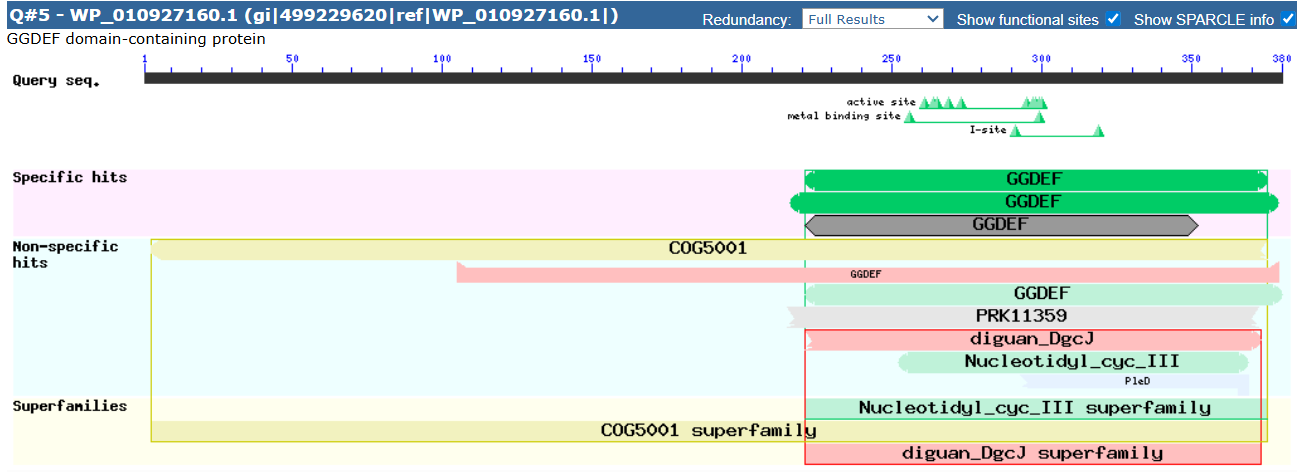


**BdcF (Bb RB50 AYT36_RS09965, BB1960, WP_010926344.1, 475 aa)**

Globin-like domain, gray; GGDEF domain, green; conserved active site motif, yellow

MKPSPEILALRWKDTCAHYSPHEWAAARNVVTANKAALADYFYECMLADPNAAFFLSDQLVKTKLHASMQDWLESVYAAAPTEEYERTVAFQRKVGEVHARIDIPVHLVMRGACALIRRICELLDRDASLSAAQAAATCRYVADVTMTAVEMMCHAYSVSHDRNARAEEGYRLLALSQNVGAERERQRAALLDWENQLMFGLSVGKPWDELPPIRKSEFGLWFIHKAAHAFEGAAESRSVSSQLQHIDQLLADAQQTQPPPDQRLAILHSVRDATKAIGFLIDGLFEQAGNLESGRDTLTRLLNRKYLHVVLSKEVAYARKTGVPLSVLVADIDYFKSINDAHGHDAGDFVLQQVASLIASYSRGGDYTFRL**GGEEF**LVVLVDADGTQALNVAEALRRRIQEKATVLPDGGEVRLTLSIGVAAHDRHPDYQRLLKRADQALYAAKSGGRNRCVLANAGPAEPSQRPDAVAQSGQH*


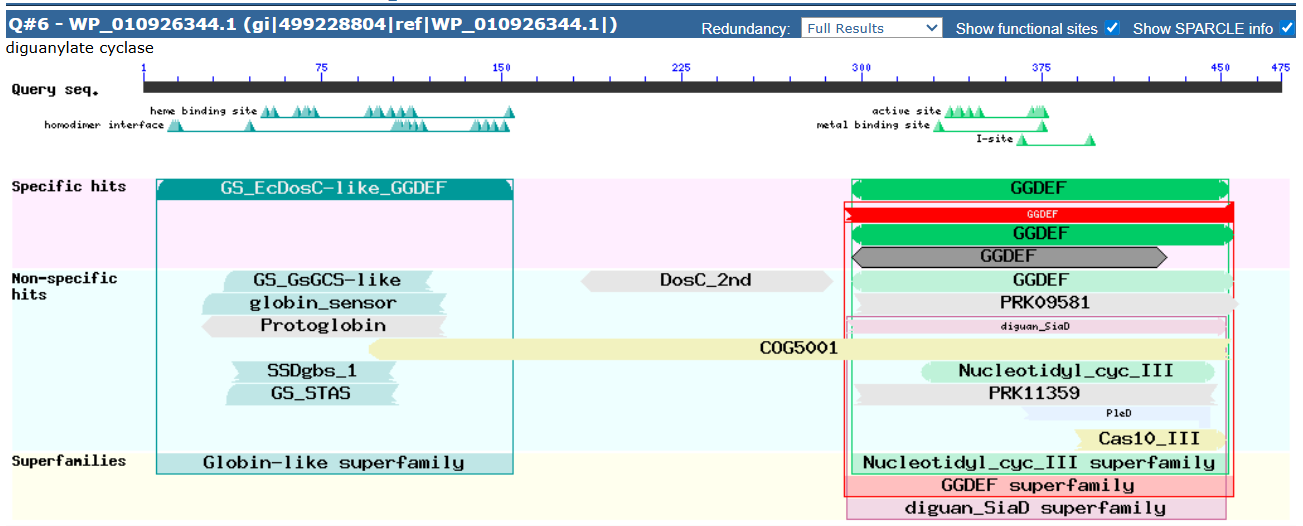


**BdcG (Bb RB50 AYT36_RS13380, BB2626, WP_003811756.1, 385 aa)**

GGDEF domain, green; conserved active site motif, yellow

MLAPVTLLVISTLSSLVTLGVLGSLLRSGIAGIRELMWANVLVLCSLLLFTLQANPAMPPWLAIQAPNVLISSGVVLFCAGVFRFMQQRPPWRLLLLGLALSIGANIWFHYVDPSVNARVVAASGLHALLWGGSAWTIYRNMPLRRSRYSYWFAGVTATVAALGHALRTAVYGLQIEQTQGLLQSSVWNVAFLAIGVLVMPSMTLGMIMMIHDRMLAEREREANQDFLTGLLSRKAWWREGERLCARALRTGRPLTLLALDIDRFKQVNDQHGHAAGDVVLRHFGSLATALLRSGDVIGRL**GGEEF**VALLPDTDGDTGVSVAERLLASVRATPCSHGGKALTYTFSGGVAQYRAGDTLPVLVERADAALYAAKQAGRDRIRNRAG*


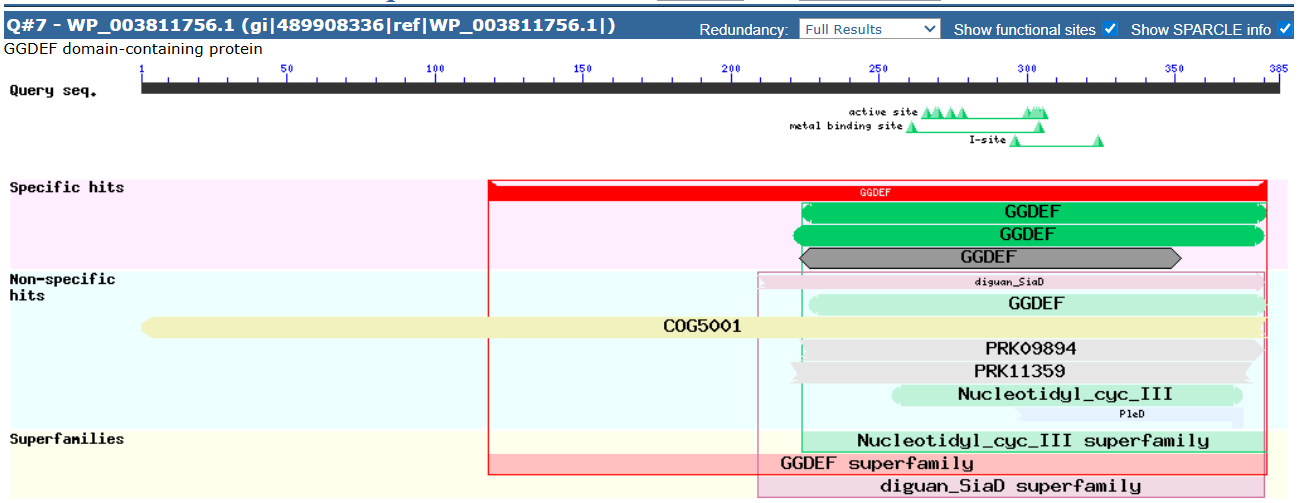


**BdcH (Bb RB50 AYT36_RS05010, BB0991, WP_010926008.1, 387 aa)**

GGDEF domain, green; conserved active site motif, yellow

MNVDLTTLYLLATGTLIASAGMMLWEYRANPGRGRALGLFAAGFATLALGCVAALYRRDLPGVWGSALANLIMIGAYLLVLQGVGALRGSRHGAFGAAVLAVMAAAWALSDPAEMDATWSHVSAAPIALINAMTALSMWRNPAMRPFPARRMVIAMTGLHALIYAGRALVLPWLVREHGLPVQALAGKFTIYEGVLYSVILPMALLRLLREEIHGELLRESQTDYLTRLGNRRWFFEEGARLLKHRPAGSSPLAVLAFDLDQFKAINDMYGHQTGDEVLQAFARAAQDVIGPRAVLARI**GGEEF**AALLRGDDALKAQALGEAVAGRFTQAVAGRLAHISLPVTVSIGLARIEPGDATLAASLMAADRAMYRAKSLGGNRLEIAAGAA*


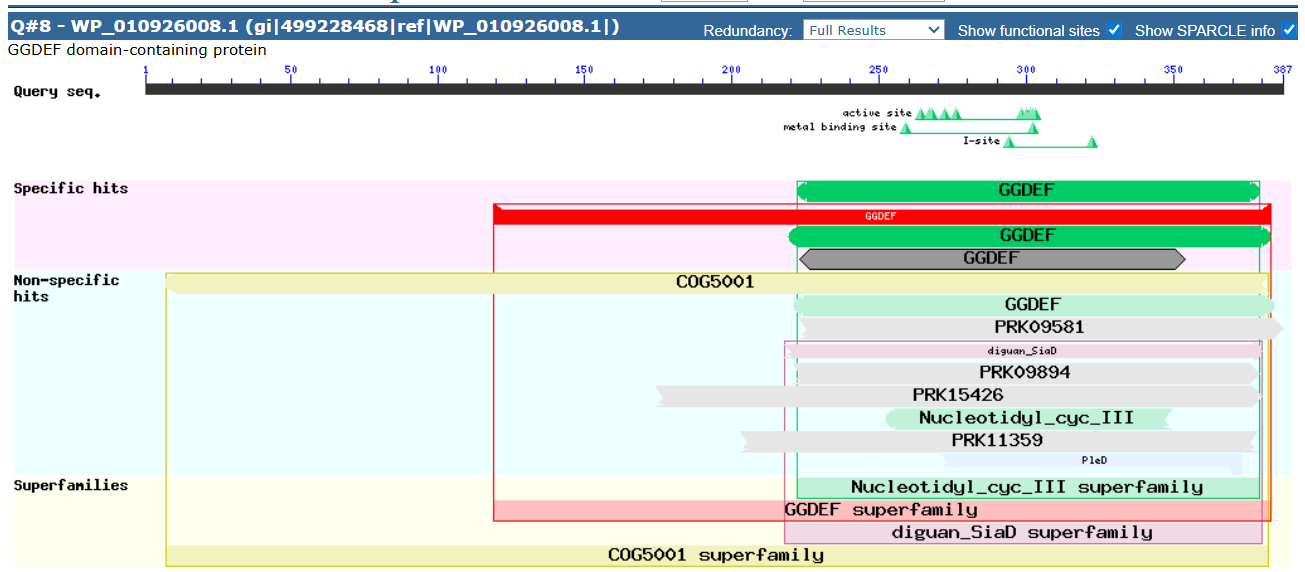


**BdcI (Bb RB50 AYT36_RS06160, BB1220, WP_003809114.1, 567 aa)**

dCache_1 domain, gray; GGDEF domain, green; conserved active site motif, yellow

MHAPWNPPILQTTPGNTTNATTSRFGGRPQRGAKNVLHNQGRRAQPMQRIVRHVTLGLALAWLALVAGLGWWISQRIVTAQLDRLAASAEYEARTTARVMDRLFTEMVSVANMVARQGQVTELAARYRTDPPGAAALTRQQRAALFTRDPLVRKVGDFMNALASDLRYARIYMNNMSDDTVTASNWAEPDSIVGMVYAGRPYLIDALRTGNGYSFGIARLNKSPSYFVASRIEDADDTPLGSVTVKFDAPEVALYLTGRHTALIVNPQGRVITASAGPFMLRNVAALLPPGSVLPPDGEEAPGEPMDVRAAGGAGRAEQWLIDGKPYLVRRQPLSGTHYRLLTLASLEHLAPLRTQHVWMTTLVAAVGLVVILLSGQAARQMVMRRQDERYAANYDALTGLPNRRAVLAELGRLFALANRLQQRVLVAFIDLDGFKSINDTYGHEIGDKFLVEAGRRMSAGLRASDTLGRW**GGDEF**VVVGLVAPSASTDPDAAAAAMRERLAPLLVGAYRFADCSFDYPGASLGVVSVDPAASSVQAVLKDADRLMYADKQARRAAGVMDGEGALPA*


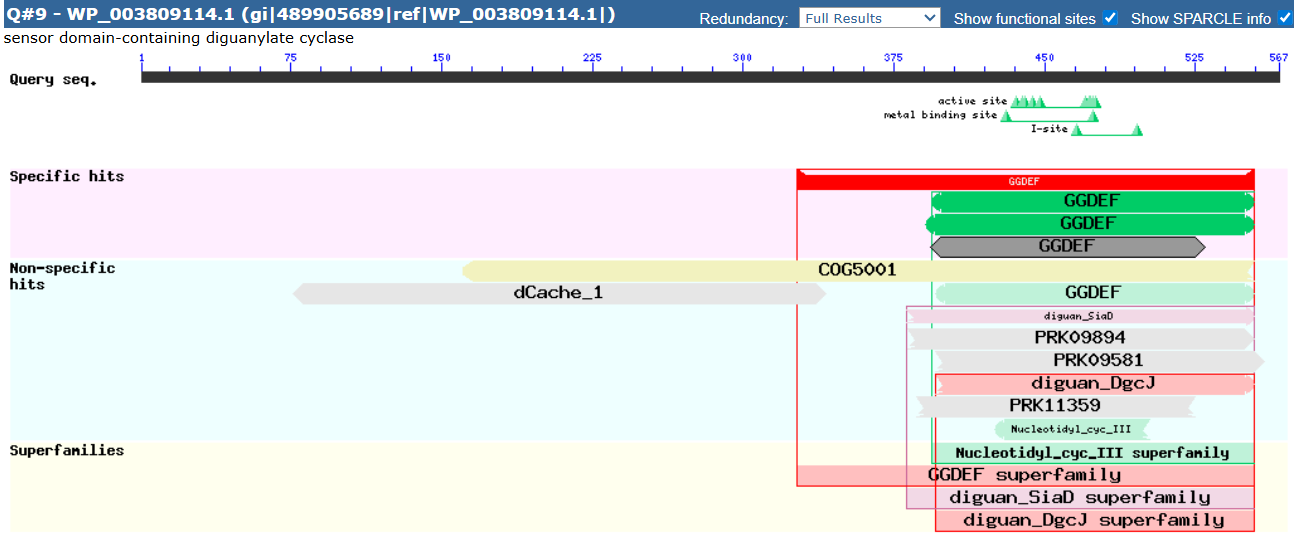


**BdcJ (Bb RB50 AYT36_RS13550, BB2660, WP_010926585.1, 500 aa)**

dCache_1 domain, gray; GGDEF domain, green; active site motif degenerate, yellow

MVTALGIVAALGIVIAVASILAQDRQQVWEREARSARNVLQALAGEFERTVTAYDLVIRGVIGDLAQPAVAAMGPEALHRLLFQQGARPDNLAVLLVLDERGEVILDSRTAVVAPRNRSRRDYFQAHVDNPQAGLFISKPYRSRNRNGELSIALSRRLAHPDGSFAGVVVAAVSLSYFRDRVVQLNVGPRGSMTLFRDDGIVISRKPYVEAQIGVDLSESANVSRFRREGRGAFVGTAILDGVERLYQFERVGKLPLIMVVALSTDDMMTPWRRHAWVLASAVLLLCALLVALLAALERELRRRRNAEAELAQLARIDTLSGLLNRRAFAQVLALEWEAALRGRTPLALLLVDVDHFKAYYDCYGRSASDVLLRRVADAVRDCVPYAANVVARH**GADEF**VVLLPGMGLRSAAWLAERVRARVLGLAVPHRGNRGGVVSVSIGCTAMTPLAGSEAAVLVERADAALYLAKSRGRNRVERIGDPSTAGQGAEREMAQEPGGP*


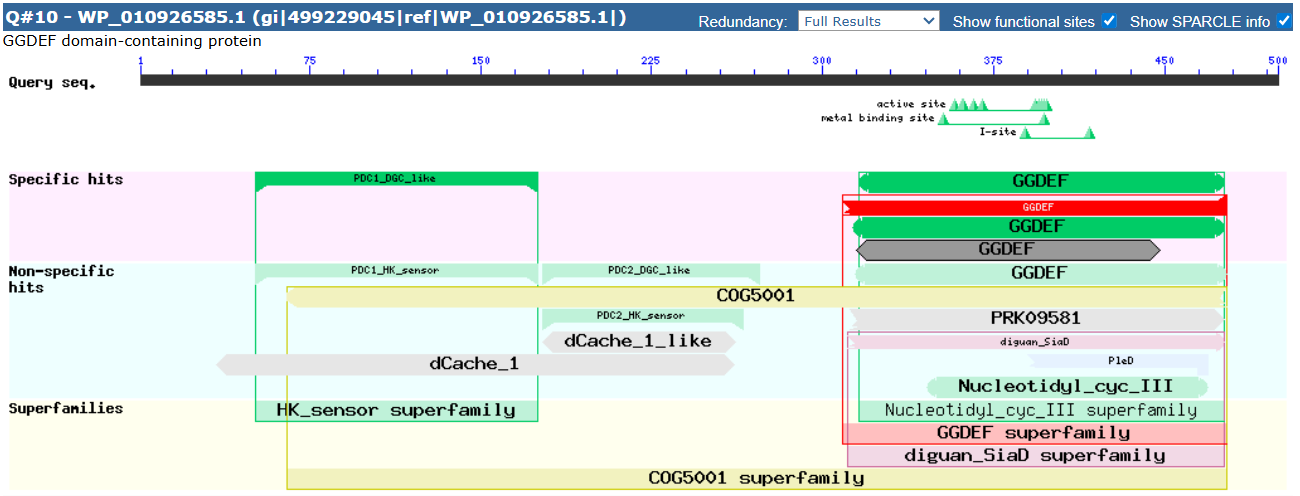


BdcJ of BppOV Bpp5 (BPP5_2486, WP_164926561.1, 592 aa) possesses a divergent and extended C-terminal portion of the GGDEF domain. The degenerate active site motif (GADEF) is identical.

**BdcK (Bp TohamaI BP1492, WP_224019411.1, 356 aa)**

GAF domain, gray; GGDEF domain, green; conserved active site motif, yellow

MAPNSKHLSLHMPVDFIDALAQAESIDEILKVVATWFMEMFVADRASITFPVNDSHLRVVALEGNRVIDVDAPVPIHGTMVGRVFSRGQAEICDDLAASTDLDCLILASRGLGSCLDAPLRSGRDCYGTINVGRRDRNGFTLADMRKIEALAMWIATLIRVHRQVERLTHLSRTDPLTQIMNRRAFTESFQTRRLEGERRRAEVGTGLGFAVVDIDHFKQINDTYGHDVGDVVLAFVGKMMNEFFRESDCVARF**GGEEF**CVLMQDVDEGGMQRLLERFRVALGDCVVTHSAGTVSITASIGAVLVKTSVAGIDRAFRSADLALYKAKADGRNRVRIAPSAEQSHAGNPANRLCYPG*


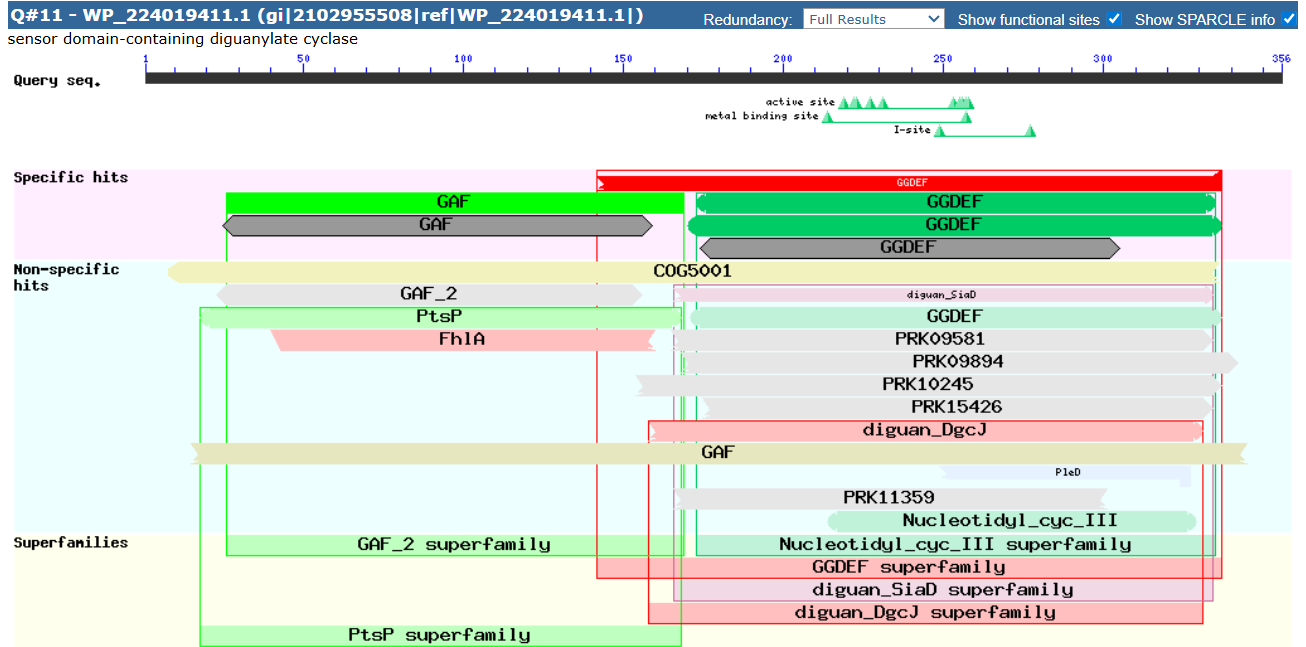


**PdeA (Bb RB50 AYT36_RS13570, BB2664, WP_003811684.1, 403 aa)**

REC domain, gray; EAL domain, brown; conserved variant active site motif (Schmidt et al. 2005), yellow

MNLPDILVVEDHPAHRLVVVHALRALGYTRILEAADGNEALRRLDEHGQVGIAICDIKMSGMDGAQFLRVAARRQLLGAVIISSDVSSDLIAAVLDMAALIGLRVLGDLAKPLDAQRLKALLDRHEAERQRARANAAPTASRQPPAPPAREVARALAAGQIVPYFQPKVDLLTLRPCGA**EVL**ARWRHPDLGVLGPASFLEALKEQDLLDRLIWHLTDAALGQARLLATAGATQDLALNFETSQLGSAELLPTLAQALRKHALPASIVTIEVTENGLLDAPAATLENLVRLRLMGCTVSIDDFGTGFSSMQRLCHLPFNQLKIDASFVRRLPGDARSESVVAATLAMAERLGITVVAEGIENPPQHQALLQLHCRQGQGYLFARPMSGPDYARWLDGAASAAAG*


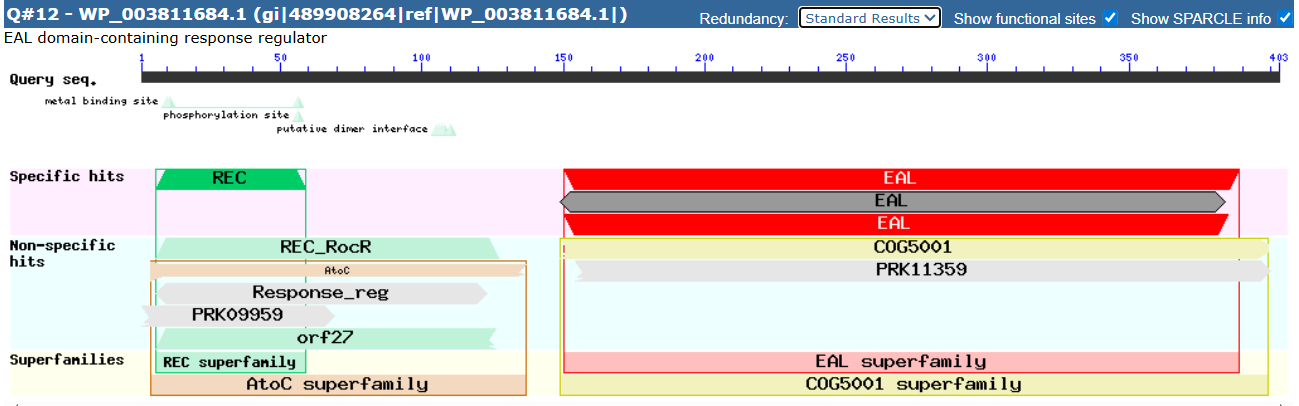


**PdeB (Bb RB50 AYT36_RS10755, BB2110, WP_003812691.1, 458 aa)**

EAL domain, brown; conserved active site motif, yellow

MGLDDLVGQWLLPGMALAAVGVFFCIKGYLLRRQMARDALTDALTVGELRRRVHALVLGKRQRVYSGAMFLVEVVHHPELSAMLGLEDNKLLLAMIAQRLQMQSDYFQVARHSSDSFMVWAPDLDPRSARDVAPEVLDALSQPYELRGQPLIVGFRLSGALYAQHGACFDELERSLHIAMLHLDESAGAAWNMFEPSMLEHQRHYQGLEHDLRVALVSSSMDQFEVCYQPVCDSVTGTIYGC**EAL**LRWHHPVHGDVSPALVIELAERTGLIVPLGAWVLETACSRAITWPAAWRLHVNVSVRQMYADELPAQVALALAASRLSAQRLVLEITESLFIQQYERHVATLNGMRAKGVRVALDDFGAGYSSLTHLRRLPIDWVKLDRGFVAEIEFDAVSREVVSALMGMCRALGLAVVAEGVETPAQREILKALGCREMQGFLLGRPVSAERIRELGAAVS*


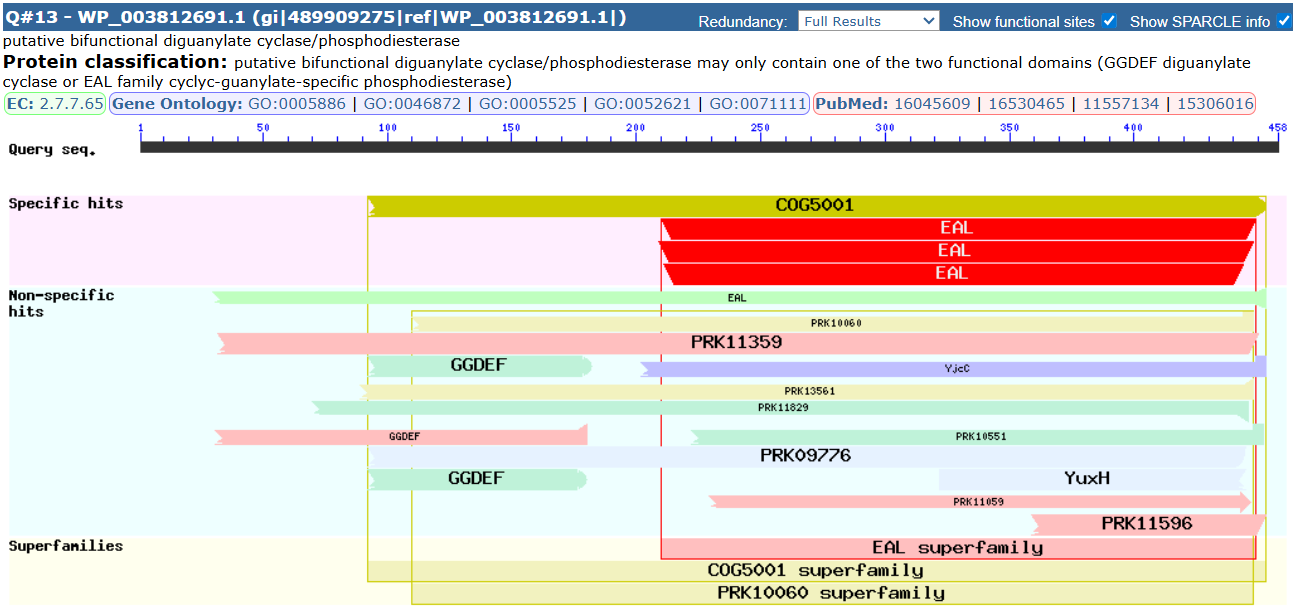


PdeB of Bp Tohama I (BP1460, WP_019247210.1, 350 aa) lacks the N-terminal 108 amino acids present in the Bb RB50 PdeB. The EAL domain is intact.

**PdeC (Bb RB50 AYT36_RS15830, BB3116, WP_225665774.1, 503 aa)**

CSS motif, gray; EAL domain, brown; conserved active site motif, yellow

MIGPSLLAVDMVREYGLRQQREQAQVLAQRLLGRIGLTADRLLRIHRELVHTGGSSPCAPYSTYLMQQQVLRFPDVRAVAYVEEGRVLCSSLGDTVTGVDLGPSDFTTGNRISVWNSAVLPAAPETPFRVFAKEGYAIIMERESTNQLGGAVPQAMAAVSFSGTPDDAIVWGELPRAWFKRARESNAFVDDKYVVAVQRSTAQGLVALGAVASSQGAEALQRLAILLVPLSAAAGLGLLVGAVILARRQFSLPQLIRSGLRRNEFYMVYQPMVEVGTGRCVGA**EAL**IRWRRRNGDLIAPDVFIDEAERVGLIGKITERVFELVSSDVQRWRGRLGDLYISINVSAADMASGAAERLLSEMFARTGLTPSQVVVELTERALLHSDEAVAAIKIIRKMGVRVAIDDFGTGYSSLAYLTSFEVDGLKIDKRFVDAIGRDAPSSSVSVHIIEMAKSLGLRVVAEGVETAGQQRFLSEHGVDYAQGWLFGRPMPLNELLAMARRPPPG*


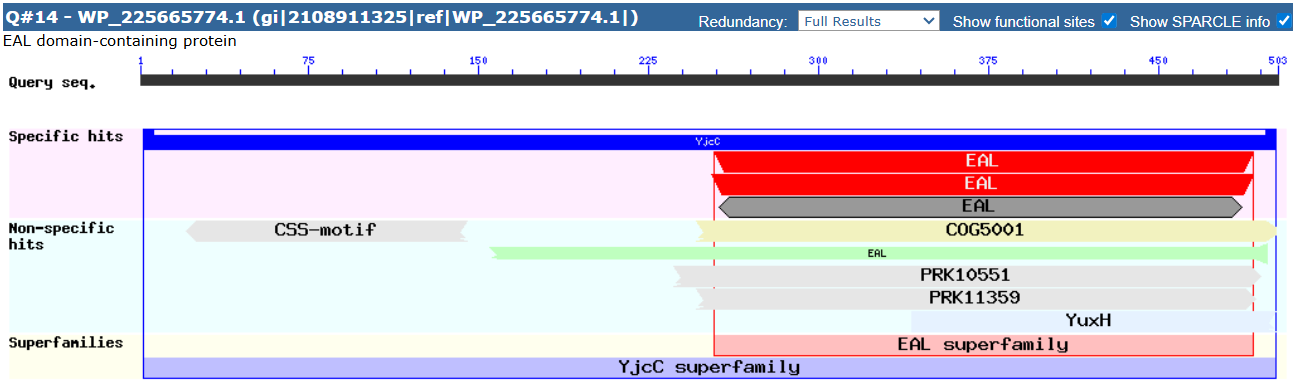


**PdeD (Bb RB50 AYT36_RS15890, BB3128, WP_225665779.1, 507 aa)**

CSS motif, gray; EAL domain, brown; conserved active site motif, yellow

MTIRYLPVRHRLLFFVVPVLACGLVAAAIGYAWKGMHATSMVEHDNAHFLARSLDIAREQLDIVLEARVAPAACSEGDLAALRQRVFRSKYVADIARIDNGALRCSAVWGVWRHPHVLPRGGRTVRDGVFLWQNMENPIQPELSGDVLASERLAVFTIPGIFDDIVRRAGGQKTTLYSRKQGHVYKVFDASAAEDWQGWGAGFQLVRTAAACAPVGGPDICVETTARVDARGAVAVAGLGGMALGAGVGLIVFLWWRRSFGLRASLVDALRKGKIQVAYQPLRVLDSGRMAGV**EAL**ARWTHDEVGPISPVTFLGWVEAMGLRQPFTRYIVQAALDGMKTRMAGDAPFYLSINVFPEDLEDESFLDFLTQSVRERGLSPGSVVLEVTESAVFSTASPSVLFRRFRAAGFRIFLDDFGVGYSNLGNIIRWEVDGIKLDRIFMQSLGDFDSAAPVLDQVIEMARQLDLHLVIEGIEVRSQADYIFKRAPHAVGQGWLFGRPGPACALPAA*


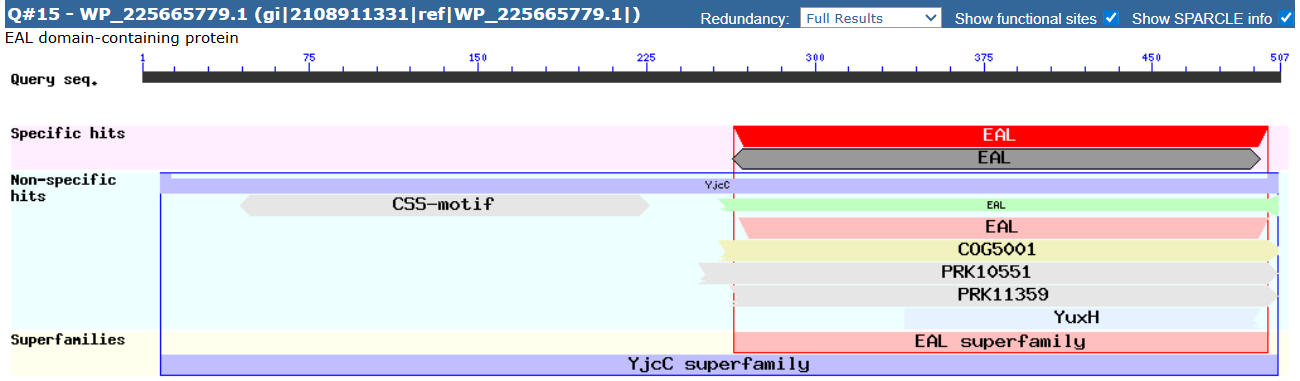


PdeD of BppOV Bpp5 (BN117_RS23995, WP_231859887.1, 309 aa) lacks the N-terminal 198 amino acids present in Bb RB50 PdeD, which constitute the CSS domain. The EAL domain is intact.

PdeD of Bpp HU 12822 (BPP2807, WP_226376560.1, 491 aa) lacks the N-terminal 16 amino acids present in Bb RB50 PdeD. The EAL domain is intact.

**BvgR (Bb RB50 AYT36_RS15225, BB2996, WP_010926709.1, 291 aa),**

EAL domain, brown; active site degenerate, yellow

MIHWTNSARSSFEASLSPSRRTLRARLAPVEGPTVAELRAALKRDEFIPAFQPVVDAGTGSLFGV**ATT**IQWAHRHWGLVPEHCYMAALVRDNLHIALAQQLLAQVFDLAAAMRRGNRHVGLFIQPEPICLMSRSYSDDVLRNLDHRGLPPFALTLAVSPSLLQSPYGQQSLHSLRRLHRAGCNLVLTVSTFDAESPPHLDALEICGIALMPELMYALRQGGRVGEACRAIAQQASAQRIPVMATGVVDEAMAQAADLLPCRYLLGDHVAPPMTGQRFLYWYYRREMPPNYP*


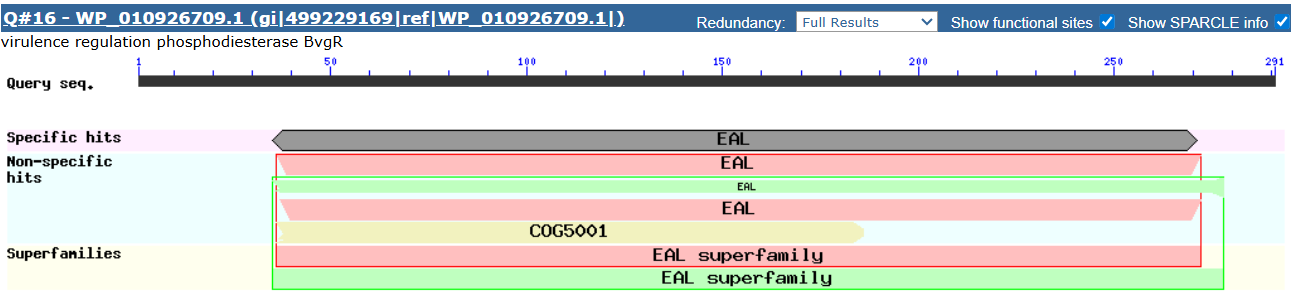


**PdeE (Bb RB50 AYT36_RS07930, BB1564, WP_003809780.1, 962 aa)**

dCache_1 domain, gray; HD-GYP domain, brown; active site motif degenerate, yellow

MAPARVCKMGNGARRLRGWPVRLLIAGLAVAAIFLVAAVLIALSWMNSRNLLLDAAARTANDAAQITFERTRRMIEPASATLRTLSFDPIVSAPRLQDRLERMRLLAEELAANPLISALYVGYDNGDFLLARMLDDPGIRQHFGAPGQARFLVQTVTRAADGTAKGDFLFYDGGLSPITRRSEADYRYDPRERPWYANAGEGAALTISAPYVFFSTRQVGVSLSRRAEGGKAIVGLDIVLDDLGRMLDELRITPSAELALVDGSGAVVAYRDPQVLTARAQAAGDTHLRPLDDLGVEPLSDLRRIARDGRPVSYDVAGHEWLGVMLPFDGFDNVDLRLLLTAPADELLGDLQHDRQRMVLITGGLILLFLGLGWWGGSRIGRALERTTAQAKRMSAFDFSRPPDAPAWLRETRELNGVMDNVSNTVEAFLAISDVLGAEPRIETMLAQVLEKFVHATRCRSGAIYLLQKDSRTMARAAVAGDAHGLEESLPCAGGGDAAPAAGAADGLQRVQFELRSRAGRVEGLLVLRHAQDQDHAAPGFQAFAFRLTGMLSAAIETRQLIESQKQLFDAVIRVLADAIDAKSPYTGGHCERVPALAIMMTDRMCADTSGPYADFTFTEDERYAFYLAAWLHDCGKITSAEHIVDKATKLEVIYNRIHEIRMRFEVLWRDAEIAHLRACLDGQPAAASAQARDERQAQLQDDFRFVAECNLGGEFLADEAIERLRRLGQATWLRHFDDGLGLAAAERRRLEGSRAGAPALPAIEPLLADKPQHRVEWGDNKPAVERDDPRNALGFDMKLPAYR**QD**MGELHNLSIRRGTLTDEDRFAINDHIVQTLIMLKQLPWPRHLERVPDIAANHHEKMDGT**GYP**RRLPGEALHLTERVMAVADVFEALTAADRPYKLPKTLSESLRIMAVMCKERHLDTELYLYFLRHRIWLAYAQQHMNPSQIDDVDIEALARIAQG*


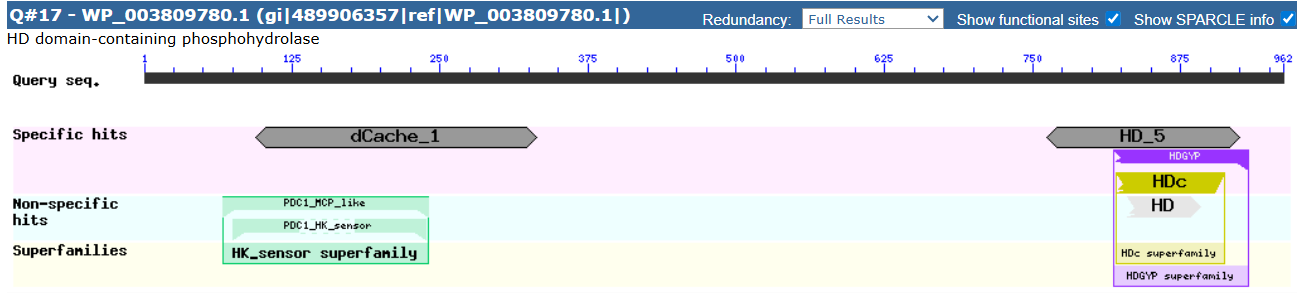


PdeE of Bp Tohama I (BP0880, WP_224019276.1, 792 aa) lacks the C-terminal 170 amino acids present in Bb RB50 PdeE. These constitute the majority of the HD-GYP domain.

**PdeF (Bb RB50 AYT36_RS09970, BB1961, WP_003813001.1)**

HD-GYP domain, brown; conserved active site motif, yellow

MQTESLPRSEADTRACRALAAALRARDPSTAIHSGRVAALAGELGRAYGLDGMAIRRLELGASL**HD**IGKLGVADRVLHHPGRLLGEDWTHMQEHSVLGERIIAATGLDDGAAIGRIVRHHHEHYDGS**GYP**DGLAGEAIPLEARLIALVDAYDAITAHRPYRPPVPHDRTMAMLEAEQGARTDPQAFTIFARMIERSQWRAG*


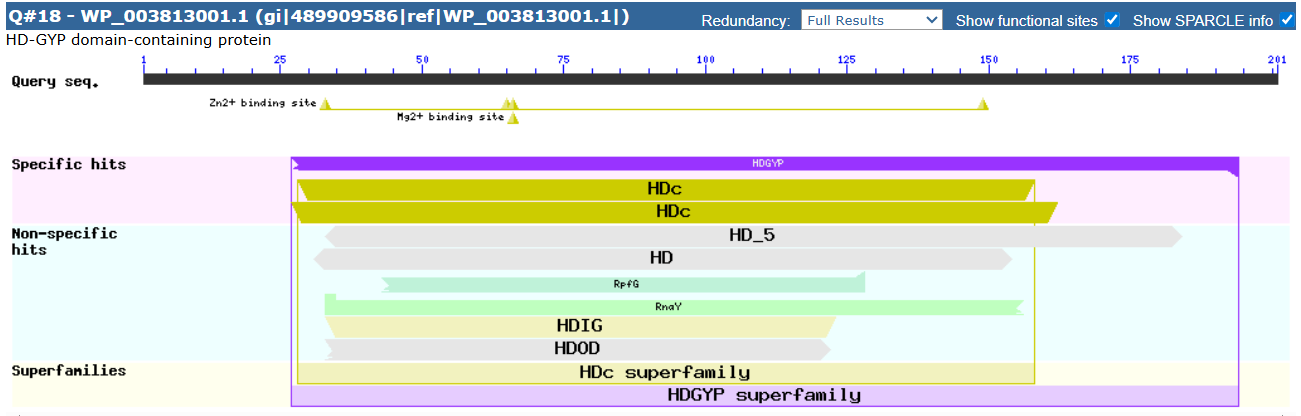


**LapD (Bb RB50 AYT36_RS05980, BB1184, WP_003809044.1, 658 aa)**

LapD domain, gray; GGDEF domain, green; EAL domain, brown; DGC and PDE active site motifs degenerate, yellow

MSILRRLLLSLTLAIGIILLGTLVLSINAARGYLSDQLQVQSTDAAVSLALSLSQPANNDPVTQELLVSALFDGGHFSLVRLADPQGAVMVERGAQPAASVPAWFQALAPLANRAASHAVSDGWRQIGEVTLVANDAYAWETLWASSLRMAALIMGAGVLWALFAFALVRWIEKRLLFQVSEQVRAIDSRASGEQAFARVAEFSGIVDALAQTRERVRATAEEQSSRIESLEVELNQDPVTGLANRKYFINEFRRALDEKPADSPALPARLSSAGGHVLVFRQRDLTALNRHMPRQFIDQWLQSVCQRISALVTAQGLAQSLVARL**NGSDF**AVLLPHCAAPQAQVIADLVRAELRTLRIPVGEGGLCRWAMALADYAPGTQFNGVLARLDFGLTRAESAGDDHAVLVGPDNADPFSAAGQSAWMDALVTALDQQRFSLAFEPLHAVDGRLVRM**EAM**LMLHNDDAREPIPAMLFIPAAVRLNLSAECDLQAVRLALDWLAAQPGELAVRLSLPSLGKRSFLRQLELMLADRRAQVGRLYLEVDAHGLVDREGDVIALERIAAHFGAHVGLRRLAQQFGAMSRLHSLPLAYVKLGGGFVGGMSQSPGSRQLAGSVIDTARALNIDVYAEDVPDAATRDILASMGVEVMRGPGVTPPPAQE*


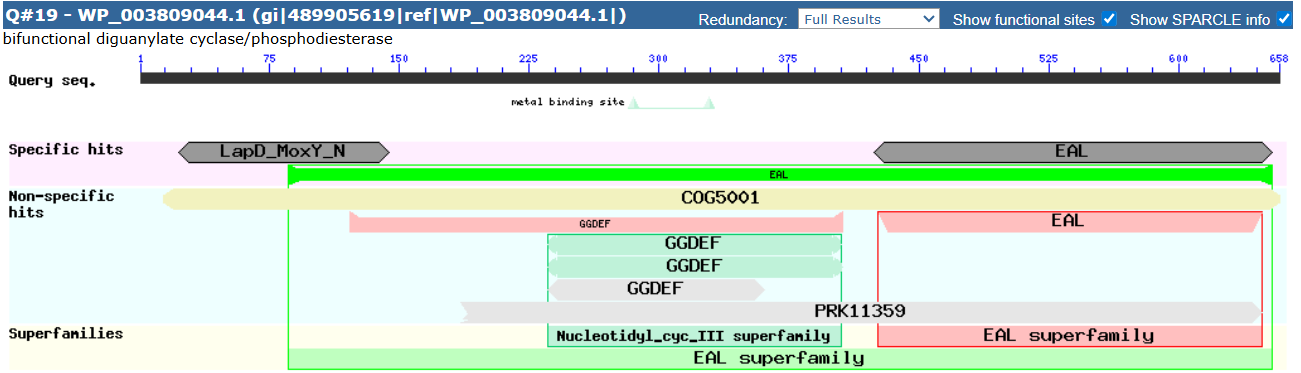


**DdpA (Bb RB50 AYT36_RS10750, BB2109, WP_033449596.1, 950 aa)**

GGDEF domain, green; EAL domain, brown; HK domain, gray; DGC and PDE active site motifs degenerate, yellow

MSISRRLFLSVVLLAGLLVIGTQAAGLAANYRASVERQKRHVETAAASLAWVLSQSHTTPAQWQTLATDLQRHGGYALVRIQEHYTGRMIEAGQLSEAPARADGSLQQGRASQIVATRTFRSADGTYVGSVAVYNPVIPILEAPFLTESVAQLVLIVAGLLIWYSYMSRLHDRLQRGPLKRLADNLAHIGQAGVDPKLAEGLPRELQPLGTALVQSHQRVHDQLRQQRARIDALENETYHDPVTRLPNRKFFNESLRRAVQRDGGVDGHLLIFRQRDMAEINRQMKREATDQWLRLACAQLSKTIKEQA**GAGAV**LVRINGSDFAALLPGLPSPRAAVLAERLRRELRVLRLPLRTHGWCRWAMALTPYTVGEQVSDILARLDHALMRAEIAESDEVEPAFSQTGNRIDGEYGWQDILTRALEQHRFFLTLYPRQDAEGQVLHT**EAQ**LTLRDQDSPEPLHAQLFMPPAARLGLSADCDIQAIRMSMDQLVARDGDMVVRVSLPSLEQAHFLKRLEDVLRDRPEQAGHLIIEIDAHGLVDYFHNVQALCEIAARTGVRVGVRRLSEQFAALERLHQLPLAYLKIGGSFVQGITRSPGNQQLADTVIQVARTLDIPAYAEDAEEPAARELLQAIGFRLMQEADNADFLAPRHNWIADLPAAAPAAAEDNGDTAAHAPTRTRATDGRPWPPHELGMQRKRQEQSDRRLAEVARALEAQRQVHALLSHELRTPAATISAAAQSLEIILAGSGQEVDSRLARIRRAVTRMIELMNQVLSPERLRDQALTPRPEPIELGELARDTVEGMRLDTAHPLVLNAEAAVPAWCDPLLTALVLRNLIHNAVKYSPADQPVHIDVGLSYGAHGATAWVAVTDRGPGISDDESQRIFEEHYRRAAHRETPGSGLGLHLARQICQSQDGTLTVQTQLGQGARFVMTLPTPGQAYTDGGNAQAANT*


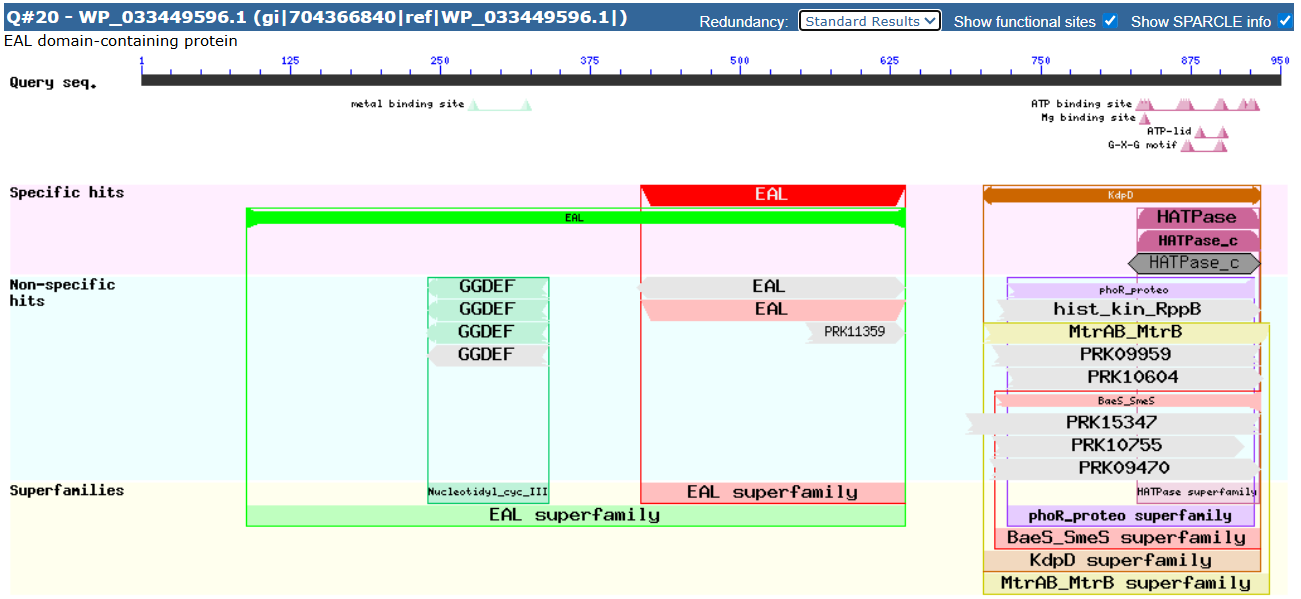


**DdpB (Bb RB50 AYT36_RS15030, BB2957, WP_003811157.1, 600 aa)**

EAL domain, brown; CBS domains, gray; GGDEF domain, green; DGC and PDE active site motifs degenerate, yellow

MISSSAADLHADAGLAAILQGGLLNPCYQPVADLAQDRIFGH**ESL**IRGPHQSVLHMPDALFAAARRDGLHAELELASLQAGARRFIELDSPGNLFLNLSGAVLVACWRHYGSGLPVMLLADSGLAADRVVIEITEYDPVGGDLPALNQALACLRASGMRVALDDYGVGNASLQLWAELAPDLVKIDRYFFNGISRDERRLQLVRSMVSTARQLNSTLVAEGIENADDLRTVYELGIRYAQGWFLGRPENEPRTDLPTAARASLQPACATQPTRIPGHTALALRVEAPPVMLARHTNDDVQRLFQEHRHLHAVAVLDTDSRPVGIINRRDFSEHYAQRYTRDLFGRNPCSTFMNPDPVLVDVHTSIDQLSHVLLSDDQRYLADGFVITRGGRYDSLGTGEALVRSVTEMRLEAARYANPLTSLPGNIPISQHIAHLLDHALDFVVCYADLDNFKPFNDVYGYWRGDDMIRLCADSIKRHCDARRDFVGHV**GGDDF**VVMLRSTDWRLRIERIIAEFNLRALDLYDDQGRHDGGIRAEDRYGVLRFFPCVTLGIGALEVSPVELGSHVRPEDLASAAARVKQQVKHGNLALLVQRYQLPARAA*


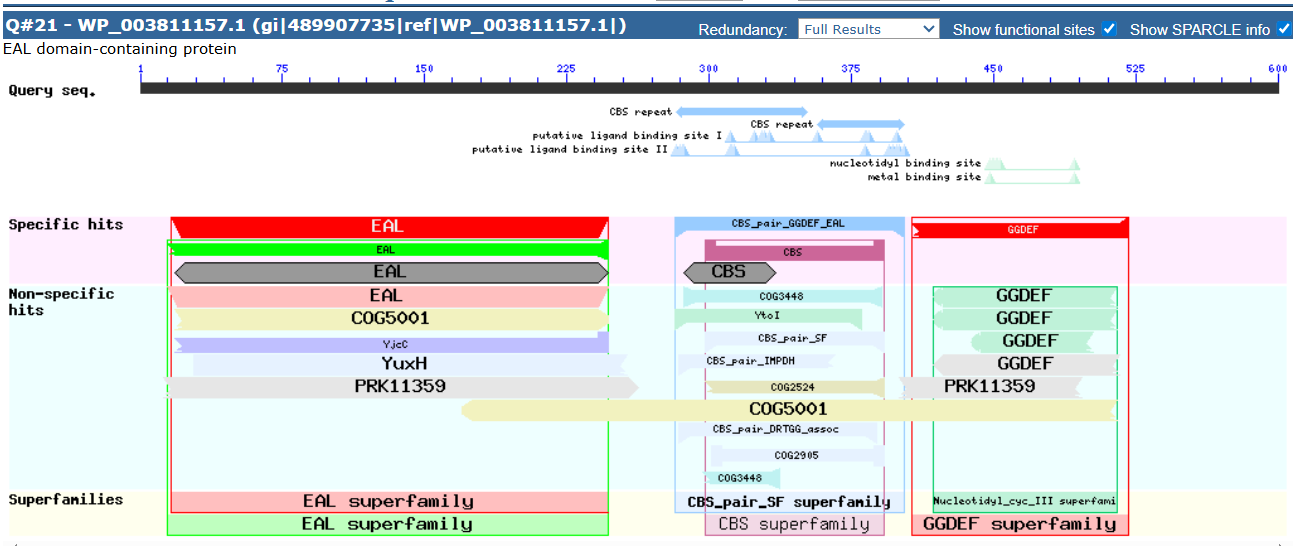


**DdpC (Bb RB50 AYT36_RS16860, BB3317, WP_010926787.1, 691 aa)**

MHYT domain, gray; GGDEF domain, green; EAL domain, brown; DGC and PDE conserved active site motives, yellow

MLVGNYDATLVLTSLFVAILASYTALGMAGRVAASRGVAARVWLGGGAFAMGLGIWSMHFIGMLAFSLPIPLGYDVALTVASMALAVACSAFALWVVTRATLPWRRLASAAALMGTGIAAMHYTGMAAMRMHPAIEYSPALFMLSVAVAIVASGAALWITHALRHNRPGAPAYRICAAIVMGLAIVGMHYTGMAAANFPQGSVCMAANSGISAGWLAVSTAAVTLAVLAVALVVAVLDTRLETRTSALNASLAQANEELVQMALHDTLTKLPNRALLDERLKQAIIRATAAGRGFSLLFIDLDGFKAINDAYGHGAGDLLLVEMAQRIKRAMRPQDCVARL**GGDEF**VVLADLPDPNDAAGLAERLIDTLTVPAEVQGHEVSVAASIGIALFPGDGADASTLLAHADAAMYHAKRLSQTHRVSFFEPSMNEDALEQLQLLQDLRLALPRNQLELHYQPKFVAPAGPVVGA**EAL**LRWNHPTRGVIGPTVFIPIAERTGLIFSIGAWVLDEACRQMRQWRDMGHADWTVAVNLSSLQFSQPDLIDSIRATLARHGLPPRCLAIEITESTAMRDAEASLAVLRELAELGVSISIDDFGTGYSSLLYLKRLPATELKIDRGFVNQLEHDNEDAAIVSAIIALGQKLNLKIVAEGVETLAQQSFLTEMGCDSLQGFLLGRPLPAAEFLAHTLQPQPA*


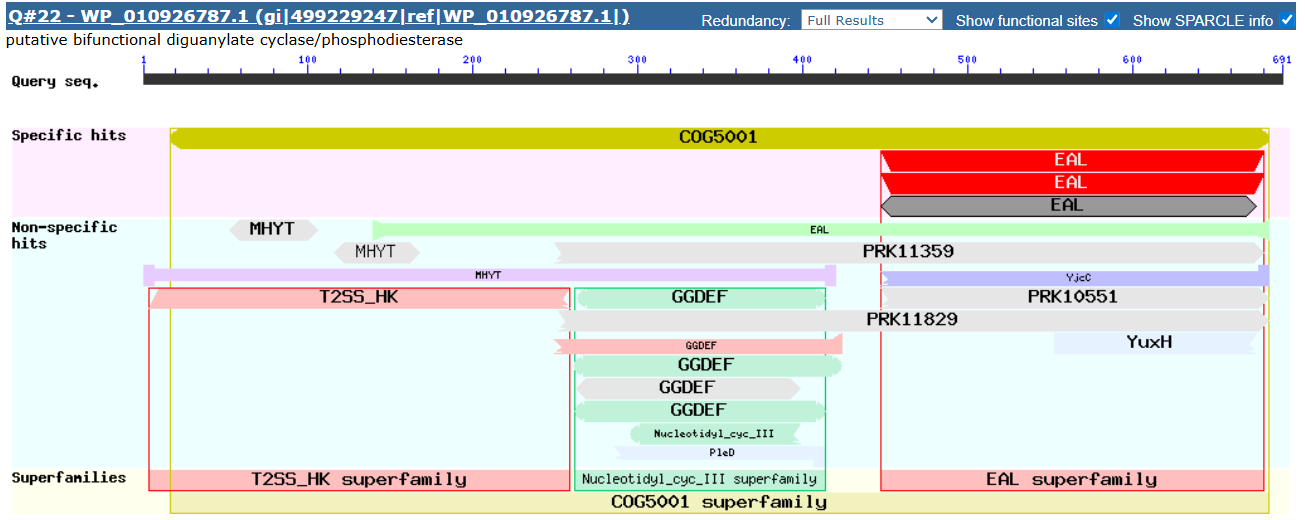

Supplement: fuaf065_Supplemental_Files [file fuaf065_supplemental_files.zip › Figure_3A_Supplementary_data.docx]
